# A phytosociological analysis and description of wetland vegetation and ecological factors associated with locations of high mortality for the 2010-11 Rift Valley fever outbreak in South Africa

**DOI:** 10.1371/journal.pone.0191585

**Published:** 2018-02-20

**Authors:** Robert F. Brand, Melinda K. Rostal, Alan Kemp, Assaf Anyamba, Herman Zwiegers, Cornelius W. Van Huyssteen, William B. Karesh, Janusz T. Paweska

**Affiliations:** 1 Cuyahoga County Board of Health, Parma, Cuyahoga County, Ohio, United States of America; 2 Department of Botany, University of the Free State, Republic of South Africa; 3 EcoHealth Alliance, New York, NY, United States of America; 4 Centre for Emerging, Zoonotic and Parasitic Diseases, National Institute for Communicable Diseases, Sandringham, South Africa; 5 NASA Goddard Space Flight Center, Biospheric Science Laboratory & Universities Space Research Association, Greenbelt, MD, United States of America; 6 ExecuVet, Bloemfontein, Free State, South Africa; 7 Soil- and Crop- and Climate Sciences Department, University of the Free State, Free State, Republic of South Africa; Nanjing Agricultural University, CHINA

## Abstract

Rift Valley fever (RVF) is endemic in Africa and parts of the Middle East. It is an emerging zoonotic disease threat to veterinary and public health. Outbreaks of the disease have severe socio-economic impacts. RVF virus emergence is closely associated with specific endorheic wetlands that are utilized by the virus’ mosquito vectors. Limited botanical vegetation surveys had been published with regard to RVF virus (RVFV) ecology. We report on a phytosociological classification, analysis and description of wetland vegetation and related abiotic parameters to elucidate factors possibly associated with the 2010–2011 RVFV disease outbreak in South Africa. The study sites were located in the western Free State and adjacent Northern Cape covering an area of ~40,000 km^2^ with wetlands associated with high RVF mortality rates in livestock. Other study sites included areas where no RVF activity was reported during the 2010–11 RVF outbreak. A total of 129 plots (30 m^2^) were selected where a visible difference could be seen in the wetland and upland vegetation. The Braun-Blanquet method was used for plant sampling. Classification was done using modified Two-Way Indicator Species Analysis. The vegetation analysis resulted in the identification of eight plant communities, seven sub-communities and two variants. Indirect ordination was carried out using CANOCO to investigate the relationship between species and wetland ecology. The study also identified 5 categories of wetlands including anthropogenic wetlands. Locations of reported RVF cases overlapped sites characterized by high clay-content soils and specific wetland vegetation. These findings indicate ecological and environmental parameters that represent preferred breeding habitat for RVFV competent mosquito vectors.

## Introduction

Rift Valley fever (RVF) is an emerging, arthropod-borne zoonotic disease [1] that could potentially be exacerbated by climate change and poses an increased health and socio-economic threat [2]. In the Republic of South Africa (RSA), livestock production contributes more than 40% to the total gross value of the agricultural sector [3]. Large RVF outbreaks were documented in RSA in 1951 [4], in 1974–1976 [5], and in 2008–2011, with most cases in humans and livestock reported in 2010 [6, 7]. Pienaar and Thompson [6] list 27 smaller, more focal outbreaks occurring between 1950 and 2011. Outbreaks of RVFV in South Africa usually occur from January to March, following the periods of high summer rainfall and temperature. The three areas of the central provinces of South Africa (the western Free State, north-western Eastern Cape and the eastern Northern Cape) are five times more prone to RVF outbreaks than other regions of the country [6]. During the 2010–11 outbreak, animal cases were first recorded at Bultfontein in the western Free State [6], and, subsequently, cases were reported from all provinces except KwaZulu-Natal. A total of 14,342 livestock cases [8] were reported on 489 farms, located primarily in the central/west portion of the Free State. Socio-economic livestock losses due to the 2008–2011 RVF outbreaks in South Africa were estimated to have cost the economy R 295.3 million [3]

The ecology of RVF virus (RVFV) during inter-epidemic periods is poorly understood. Mostly based on a study by Linthicum et al. [9], it is generally accepted that *Aedes* spp. transmit the virus transovarially and the desiccation resistant eggs can survive long enough to maintain the virus between outbreaks [10]. It appears that a low level of RVFV transmission occurs during inter-epidemic periods. Activity of RVFV without noticeable outbreaks or clinical cases has been reported in African wildlife [11, 12, 13, 14], cattle [15], sheep and goats [16], and in humans [17, 18], based on serological surveys.

The association between abnormally high rainfall and RVF outbreaks has been documented by Anyamba et al. [19] and Sindato et al. [20]. Weather variables and land use/land cover have been shown to have a direct correlation with increased breeding of certain mosquito species [21]. The rainfall across South Africa was abnormally high in 2010 [19, 22], creating favourable breeding conditions for *Aedes* and *Culex* mosquitoes.

Prior to this study, no phytosociological investigations had been undertaken to explore the relationship between landscape-level ecology and reported RVF cases in livestock. This study compares wetland vegetation [S1 Appendix, Synoptic table] and ecological conditions with those described for Kenya by Linthicum *et al*. [9] and, more recently, by Arum et al. [23] which listed 9 plant species used by mosquitoes as resting sites. In Tanzania, Sindato et al [20] investigated the potential effects of temperature, precipitation, elevation, soil type, livestock density, rainfall pattern, proximity to wild animals, protected areas and forest on the habitat suitability for RVF occurrence. However, the study did not investigate wetlands, or wetland vegetation and the association with floodwater *Aedes* species and sites of high RVFV activity.

The aims of this study were to: 1) conduct wetland vegetation surveys at sites of high RVF case mortality rates in livestock during the 2010 disease outbreak in the Republic of South Africa; 2) collect, describe and analyse biotic and abiotic environmental samples; and 3) combine the vegetation and environmental data to better understand the landscape-level ecology of RVFV.

## Material and methods

**Fig 1:**
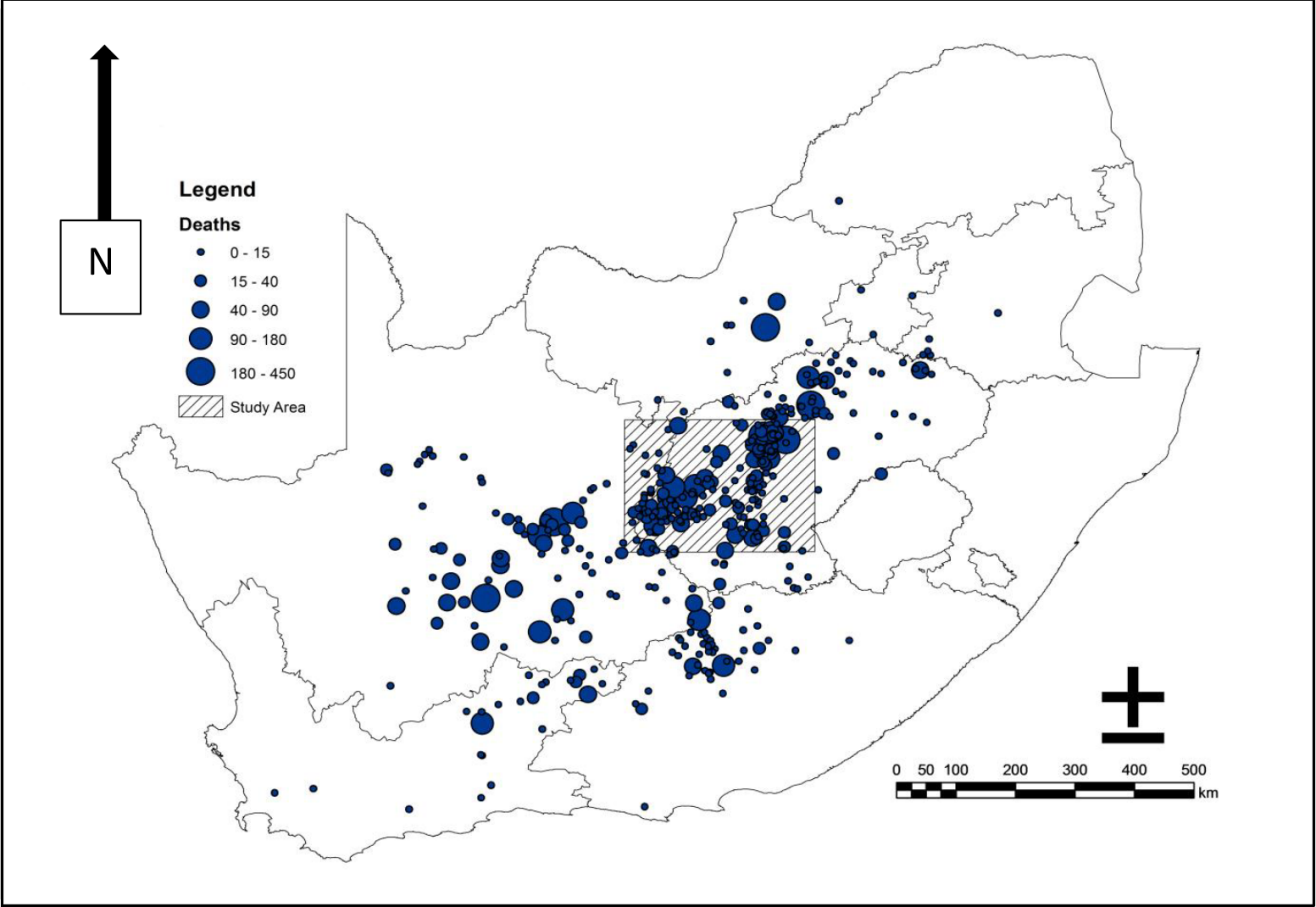
Reported deaths of livestock due to Rift Valley Fever, during the 2010 outbreak, centred in the Free State, South Africa. The diagonally lined box indicates the 200km x 200km study area. Rift Valley Fever deaths of livestock were reported in eight of the nine South African provinces. The study area was centred in the region of highest mortality in the western Free State. Mortality data derived from the RSA, OIE Report 17.

The 2010 RVF outbreak occurred over a wide area of South Africa (Fig 1). The study region covered a distance of 100km north and south of Bloemfontein and 100 km west and east of these points (Longitude 24.2000 E to 26.4000 E, Latitude: 28.2000 S to 30.2000 S), with a total area of 40,000 km^2^ (Fig 1). In the interior of South Africa, it is estimated that there are over 10,000 pans, with the greatest number in the Free State [24, 25]. Pans are regarded as land-types from which there is no drainage [25], which is an important environmental parameter for the breeding habitat of floodwater *Aedes* mosquitoes.

### Site selection

**Fig 2:**
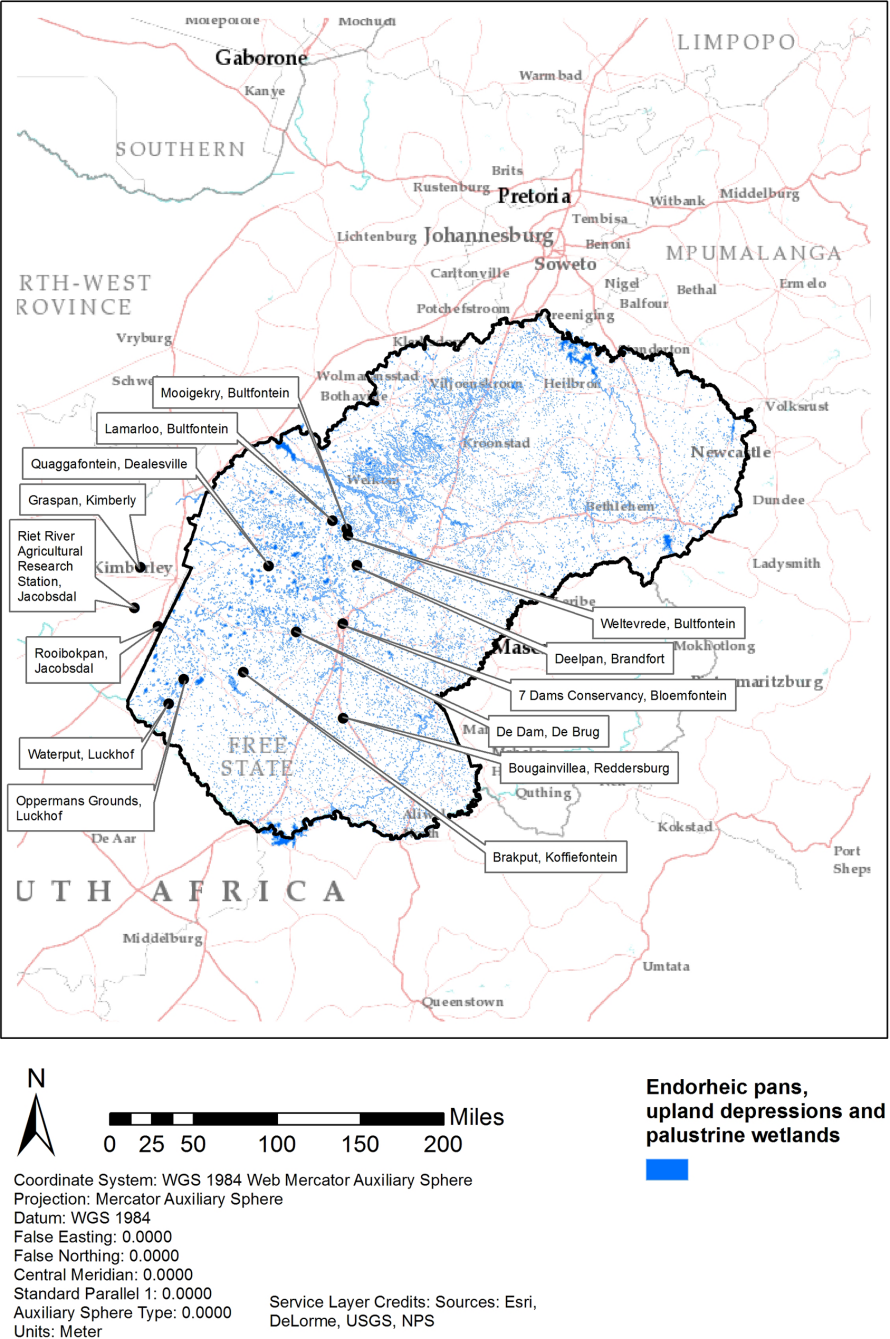
Location of study sites and nearest towns, with endorheic pans, upland depressions and palustrine wetlands shown in blue. Study sites are situated in the areas of highest Rift Valley Fever mortalities coinciding with the most dense concentration of wetlands in the western Free State.

Fifteen sites were selected. Five sites were selected at 40 km intervals along an East-West transect from Bloemfontein to Mokala Nature Reserve regardless of whether livestock mortality was reported due to RVF (Fig 2). The remaining 10 sites were selected based on locations with reported RVF mortalities in livestock in South Africa [8]. Sites were selected with the farmer’s permission and were based on the size and type of wetlands and associated vegetation on each farm. Each of the final 15 study sites were assigned a unique code which includes the farm name and nearest town e.g. Brakput, Koffiefontein (S2 Appendix, Site ID code e.g. p005petbrkp for Koffiefontein).

### Geology, soils and land-types

Underlying most of the study sites are the shales and sandstones of the Karoo Supergroup. The Karoo Supergroup extends from South Africa into Zimbabwe, eastern and central Tanzania and a small portion of Kenya [26]. These sediments were emplaced over a 250 Million period and, in South Africa, capped by a 1000m thickness of the Stormberg lavas. The endorheic pans and upland depressions larger than one hectare are believed to be the remains of a tectonically disrupted palaeo-river system [26, 27], which occurs throughout the study area. The shallow, upland depressions less than one hectare may be the result of aeolian deflation, salt weathering or animal hoof-related depressions [28, 29]. Whatever the geomorphological process, these wetland systems were the sites of highest mortality during the 2010 RVFV outbreak, (Fig 1 and Fig 2). Topography of the study area is relatively flat with dolerite mesas and low hills characteristic of the Free State. River systems are mature with meandering stream-beds and numerous oxbow cut-off streams. The Ecca and Beaufort shales and sandstones weather to produce grey, high clay-content soils.

### Rainfall

**Fig 3:**
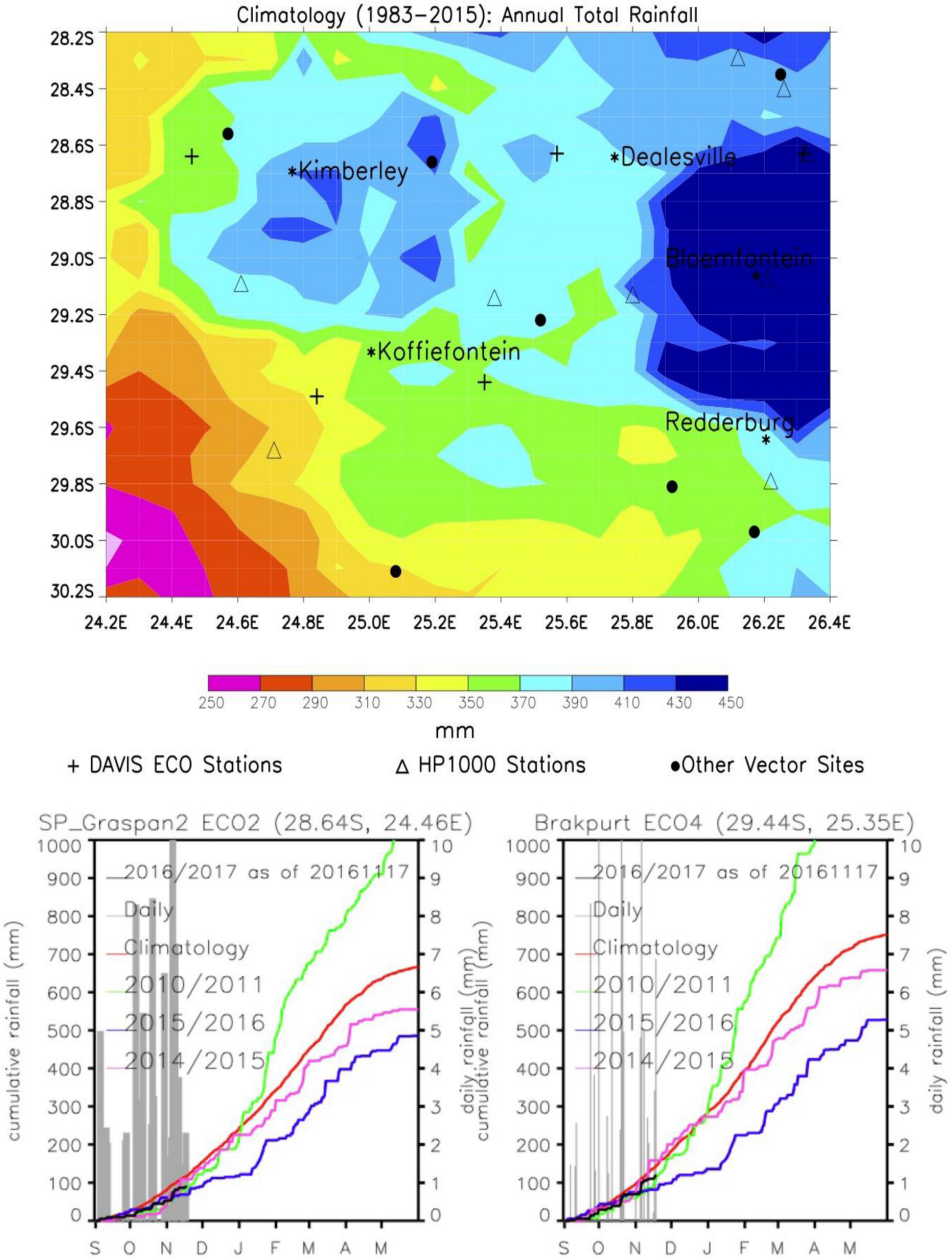
Rainfall data for the study sites. Fig 3a. Annual total rainfall map for the study region showing a gradient on decreasing rainfall from East to West/Southwest. Also shown are locations of weather stations (that are coincident with vector sampling sites) and other vector sampling sites at farm locations with high mortality during the 2010/2011 epizootic outbreak. Fig 3b. Cumulative daily rainfall profiles for Graspan/Holpan and Brakput monitoring locations. Graspan and Brakput locations showing rainfall trajectories for different years including the RVF epizootic year 2010/2011 (above normal rainfall shown in green) and the record drier-than-normal year 2015/2016.

The interior of South Africa in which the 2010 outbreak occurred, is arid to semi-arid with precipitation ranging from 450 mm in Bloemfontein, to less than 220 mm in the far west [30, 24]. Spatial rainfall distribution derived from the Africa Rainfall Climatology data set [31] has been recorded daily, and presented in graphs showing cumulative daily rainfall comparisons for different years from September to May for two selected vector sampling locations (Graspan/Holpan Nature Reserve and Brakput Farm) with the current rainfall slightly below normal (Fig 3A and 3B). Rainfall climatology is derived from Africa Rainfall Climatology (ARC) created by the National Oceanic and Atmospheric Administration- Climate Prediction Center (NOAA/CPC) [31].

As shown in Fig 3B, the year of the 2010/2011 RVF outbreak was wetter than normal at the two locations (green line), with the cumulative long-term mean (red line) of ~600mm. Such rainfall conditions as in 2010/2011 result in widespread flooding of pans, enabling the emergence of large populations of mosquito vectors and increasing the potential for outbreaks [9, 19, 5]. Subsequent years have been drier than normal, including the record drought year 2015/2016 (blue line). Most of the pans were dry with few or no mosquitoes collected at most sampling locations. Mosquito populations, including potential vectors of RVFV, increased in the following 2016/2017 summer. Field trips were conducted to look for and collect adult mosquitoes, pupae and larvae from December 2014 through to March 2015 at all sites except Lamarloo and Bultfontein (p004bullmrl).

### Vegetation and wetland survey

The study sites occur in the Grassland Biome [24] which comprises most of the Free State and covers areas of previous high RFV mortality [5, 8]. Fieldwork was carried out from October 2014 to March 2015. The study used the modified Braun-Blanquet scale [32] and surveyed 129 transects with a minimum of 4 plots per wetland [33], (including two additional sites where sampling had to be stopped due to inability to re-access the sites).

Selection and sampling of vegetation was conducted where a visible difference was seen in wetland vegetation found on shallow, isolated, non-saline depressions, littoral zones of open pans, and anthropogenic or riparian areas. Sample plots were located on a random basis within these units to ensure all vegetation variations were accounted for [34]. The presence of hydrophilic vegetation in the different plant communities was determined using the dominance ratio method [35, 36]. The wetland community types were also defined using the “association concept” which states that floristic composition resulting from certain environmental conditions (soil and water amount/depth) display relatively uniform physiognomy [35, 37]. The term “dominant species’ used in the descriptions refers to those species with the highest percentage of canopy cover [34].

All plants were identified, vouchered, pressed and labelled according to standard, botanical field-techniques [38], a full species list with authors and voucher numbers is given in S3 Appendix. Initial identification of plants was conducted in the field, and confirmed in the Geo Potts Herbarium (BLFU) at the University of the Free State which houses the authenticated voucers. Challenging or unidentified material was confirmed at the South African National Biodiversity Institute Herbarium in Pretoria (PRE). Plant species nomenclature is according to Germishuizen et al. [39], and updated with the March 2014 PRECIS database at SANBI, Pretoria.

### Abiotic environmental samples

Environmental samples included soil and water. Water temperature was taken using a standard laboratory mercury thermometer (range 100–0°C) on the surface of the water and at a 15 cm depth. Ad hoc, in-sun, ground, spot-temperatures were taken on various substrates using an infrared, hand-held thermometer (Major® MT 691 InfraRed thermometer).

### Data collection

Plot sizes varied according to wetland type, location and physical access to sites, and were estimated at 6 x 5 m^2^ or 10 x 3 m^2^ to total 30 m^2^ to comply with theoretical criteria [40] and established field practice in South Africa [41]). In all sample plots each species was recorded, all plants counted and cover was estimated using the modified Braun-Blanquet cover/abundance scale; r, +, 1, 2a, 2b, 3, 4, 5 [33, 42, 43].

Habitat and floristic data was captured using VegCap [44], with the subsequent relevés generated exported as a Cornell Condensed format file (CC!) into Juice version v7.0.28 [45]. The raw VegCap data is presented in S4 Appendix.

### Data processing

An initial approximation at clustering was conducted using TWINSPAN (Two-Way Indicator Species Analysis) algorithm of Hill [46] using Juice version 7.0.28 [45]. The synoptic table was produced; separators were defined at six hierarchical levels, fidelity was calculated using the phi coefficient, which considers only presence/absence data to reduce the subjectivity of the cover/abundance method as discussed by Lepš and Hadincová [47], with group size standardised. The diagnostic species were identified by a statistical fidelity measurement [45]. The Fisher’s exact test was employed along with the phi coefficient fidelity measure to calculate the true probability of obtaining the observed number of occurrences of the species in the vegetation unit under the null hypothesis of independence. Using the two tests together ensures that values that are not statistically significant at the predefined P-value (<0.001) are assigned a fidelity value of 0. The Braun-Blanquet normal scale was used, and a combination of frequency, fidelity and cover was selected using the default settings of 67% frequency and 45.3% fidelity. Despite the subjectivity and inaccuracy of the Braun-Blanquet method and the use of non-numerical scores ‘r’ (rare) and ‘+’ (present in low numbers with no cover), which pose computation problems as discussed in detail by Podani [48], this method of field data collection was used to conform with, and make this survey’s data compatible with the thousands of relevés already sampled in South Africa, e.g., Brown et al. [41] and Brand et al. [34].

### Classification of wetland species

The wetland-indicator status of plants (the degree to which plant species associate with wetlands) was used [36, 49], which categorises wetlands as follows:

Obligate Wetland (OBL): estimated probability >99% in wetlands.

Facultative Wetland (FACW): estimated probability 67% - 99% in wetlands.

Facultative (FAC): estimated probability 34% - 66% in wetlands.

Facultative Upland (FACU): estimated probability 67% - 99% occur outside wetlands, occasionally found in wetlands (estimated probability 1% - 33%).

Obligate Upland (UPL): estimated probability >99% occur outside wetlands.

### Wetland vegetation classification

Within Juice the lower threshold values for the diagnostic, constant and dominant species when applying the 'Analysis of Columns of Synoptic Tables' [45] function were set to 70, 60 and 50 respectively, while the upper threshold values were set to 80, 70 and 60. Species that exceed the lower threshold are listed while those that exceed the upper threshold are printed in bold in the Juice table.

### Naming of plant communities

The naming of plant communities was done according to the standard system in current use in South Africa and according to the guidelines suggested by Brown et al. [34]. The synoptic plant associations presented use community, sub-community and variant, which are analogous with alliance, association and sub-association, the original hierarchical designations used by Braun-Blanquet [32], and discussed by Westhof & van der Maarlen [40]. The syntaxonomic names for the communities, sub-communities and variants were derived according to diagnostic, dominant and constant species obtained from floristic and environmental data processed in Juice [45].

### Gradient analysis

To achieve a normal distribution, the species data were log-transformed during ordination [50]. Groups of similar ecological characteristics were identified and related to environmental gradients. A final manipulation of relevé columns and species rows was done in Juice to fine-tune the phytosociological table, which was exported into Excel and refined for presentation by moving rows containing species and adding alphabetic letters to denote species groups (S1 Appendix). The synoptic/syntaxonomical table presented as S1 Appendix, is the basis of the phytosociological analysis and description. For verification and authentication, a list of all plant species collected with authors, is presented in S3 Appendix, and curated by the Geo-Potts Herbarium, University of the Free State.

## Results

**Fig 4:**
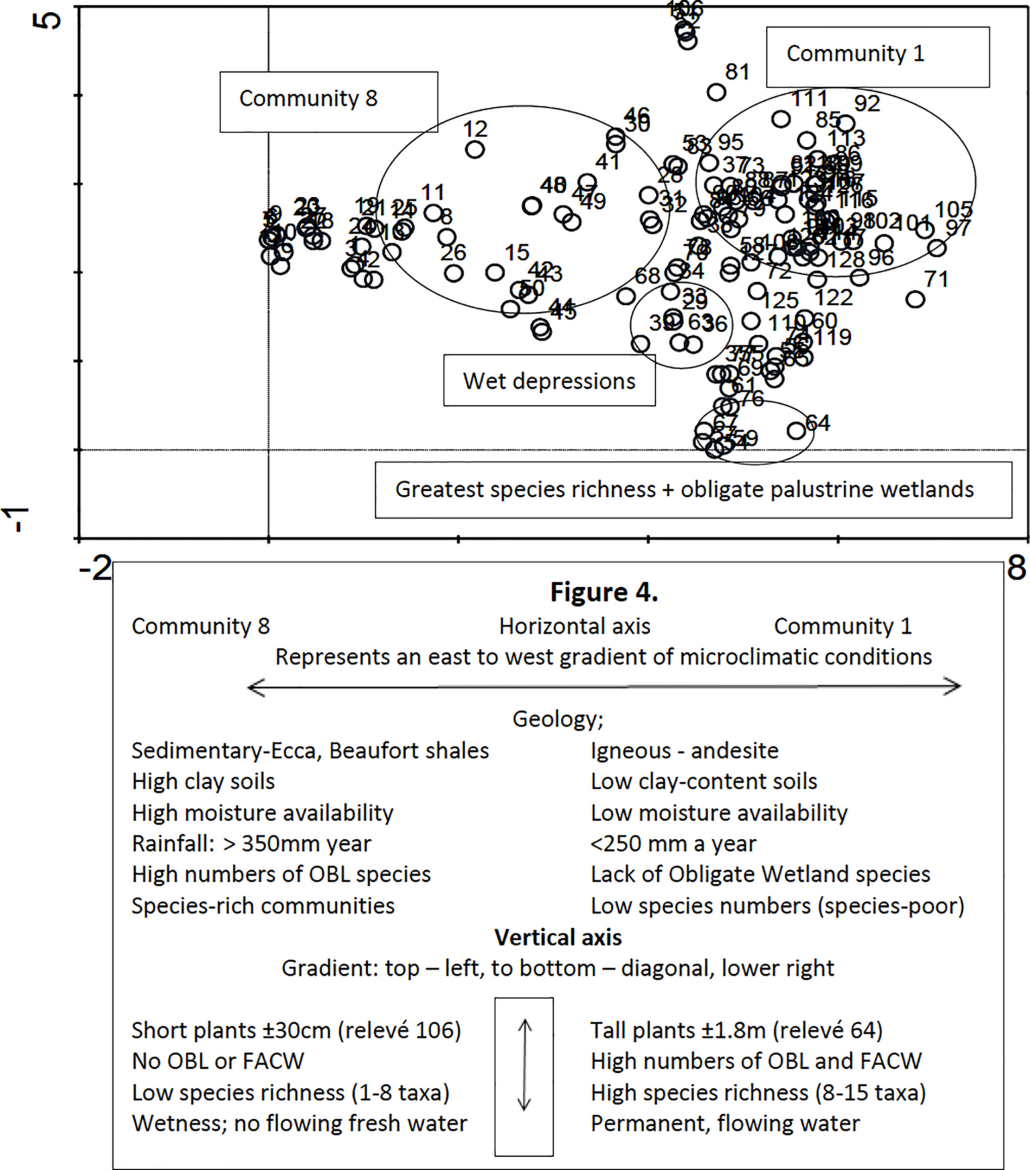
Ordination: The ordination diagram illustrates the gradients of ecological and microclimatic conditions. Community 8 to Community 1, horizontal axis represents an east to west gradient of geology, solid, species diversity. Vertical axis shows plant height, wetland status and degree of wetness.

The skewness and kurtosis calculations performed with PC-ORD v5.0 revealed the non-unimodal distribution of the species data (also confirmed by the disjunct nature of the dataset as indicated by the Detrended Correspondence Analysis (DCA) eigenvalue of one for the first axis [51, 52]. The Detrended Correspondence Analysis (DCA) produced the Ordination Diagram (Fig 4) which shows the relationship of the identified plant communities with the environmental variables.

The analysis of the 129 relevés using TWINSPAN incorporated in Juice produced 11 clusters. A final manipulation of relevé columns and species rows was done in Juice to fine-tune the phytosociological table, which was exported into Excel and refined for presentation by moving rows containing species and adding alphabetic letters to denote species groups (S1 Appendix). The synoptic table shows 9 significant clusters comprising 8 communities, 7 sub-communities and 2 variants. Two clusters are single relevés and do not contribute significantly to the wetland classification and description. Consequently, they have been moved to the extreme right of the synoptic table. A full description of plant communities is given in S1 Appendix “Syntaxonomic description of plant communities and analysis of ecological parameters”, and should be read in conjunction with S5 Appendix.

### Ecological and microclimate patterns

Of the three ordination diagrams configured in Juice, the ordination diagram (Fig 4) has the highest values for the horizontal axis, with the vertical axis the next strongest. Relevés 81, 111, 92, 85, 113, 105, 97, 101 clustered to the upper-right of the ordination diagram and relevé 106 (short plants<30 cm tall) indicate a gradient of plant-species found on low-saline soils, in arid areas with low rainfall, which form Community 1. Relevés 81 and 52 are outliers of Sub-community 8.1 with high cover/abundance values for the sedge *Cyperus marginatus* but have low species numbers as do most of the relevés comprising community 1. The gradient also indicates over-all low species numbers in response to low-rainfall, i.e. arid conditions and with low clay-content soils. An interpretation of the ordination diagramme (Fig 4), is enhanced by knowing the ecology and botany of the study sites.

Relevés 12, 11, 8, 21, 19, 25, 48, 47, 49, 30 on the vertical axis of the ordination diagram, display a gradient of saline soils and associated halophytic species found at/on endorheic salt pans comprising community 8. Relevés 39, 63 and 36 indicate a gradient for upland, wet depressions with relevé 63 (Community 3) with 5 OBL species. Relevés 59, 54, 57, 67, 76, 64 on, or close to the horizontal axis, constitute a gradient indicative of semi-permanent to permanent inundation by fresh water, on low clay-content soils of low salinity. The outlier, relevé 64 has the two, tall, OBL species *Typha capensis* and *Phragmites australis* plus 5 additional OBL and 3 FACW species, and with relevé 76, which was composed of 12 species of medium to tall sedges, grasses and rushes, are indicative of micro-ecological conditions of palustrine wetlands. These relevés are from Community 2, 7 and 6, and all have high species richness, indicative of a gradient located at 7 Dams (S2 Appendix, p014blo7dms).

The outlier, relevé 71 (Community 6) has low species numbers and only two FACW plants unlike the rest of Community 6, defined by the 12 members of species groups P and Q (S1 Appendix), of which 8 are OBL or FACW species. It is associated with Community 6 due to its high cover/abundance of *Cyperus longus* (2), along with *Agrostis lachnantha* (2) which comprised, respectively, the dominant and diagnostic species for this community. The tight clustering of relevés 68, 72, 125, 58 and 122 at the centre of the diagram represents the 3-D overlay of Communities 6, 3 and 7 respectively illustrating the gradient of wetness represented by the numbers of OBL and FACW species decreasing to both the right and left.

### Floristic composition

Of the plants collected, 34% where Monocotyledons and 66% where Dicotyledons. There are 79 species that occur in ≤ 4 relevés and which do not form appreciable clusters, and have been left out of the formal phytosociological classification and description. However, as some of these may be habitat specialists, or endemics, all species are included in the synoptic table, S1 Appendix, and the full species list in S3 Appendix.

Of the 158 plant species identified (S3 Appendix), 60 are graminoids (sedges, grasses, rushes and bulrushes) and 108 are forbs or sub-shrubs. Despite Poaceae having the greatest number of individual species (36), grasses form a less-significant component of the wetland vegetation. The two medium-size grasses; *Agrostis lachnantha*, *Sporobolus albicans* and the low, spreading *Cynodon dactylon*, are dominant and diagnostic species. Sedges, Cyperaceae (21 species), comprise the most significant and dominant vegetation type, with the medium-height *Cyperus laevigatus*, *C*. *longus*, *C*. *marginatus*, and the tall, sharp-tipped *Scirpoides dioecious* the most important. Juncaceae, with *Juncus exertus* and *J*. *rigidus* forms a dominant component of about a third of the vegetation (S1 Appendix). Of the 21 sedges, only three genera, *Cyperus*, *Carex* and *Schoenoxiphium* have the triangular stem, a distinctive characteristic of most other species of Cyperaceae.

The wetland vegetation is dominated by sedges (Cyperaceae), then rush’s (Juncaceae), and thirdly, grasses (Poaceae) (S1 Appendix). Non-graminoid plants, forbs and sub-shrubs, form a lesser vegetation component, with the most wide-spread forb, *Pseudognaphalium luteo-album*, a thin, grey, pubescent Asteraceae. The vegetation and species composition (S1 Appendix) shows a gradient from west to east, with only, low to very-low, spreading grasses constituting the dominant cover with a complete absence of *Juncus* and almost all Cyperaceae forming the vegetation of Community 1. Communities 2 to 7 all have sedges and grasses as dominant and diagnostic species. Numerous Facultative and Obligate Wetland forbs comprise significant species composition with Community 5 comprised of the completely submerged aquatic plant *Lagarosiphon major*. The vegetation gradient is also indicative of soil-type, and its change from west to east, as well as overall wetness. The vegetation comprising the palustrine freshwater wetlands at Seven Dams in Bloemfontein is composed of a mix of sedges, grasses, *Juncus* and forbs, including several geophytes, and has the highest species richness. The wetlands are located on dolerite with low salt-content soils. No livestock mortalities were reported during the 2010/2011 RVF outbreak from Seven Dams.

The small, 4-leafed, clover-like fern *Marisela capensis* is an OBL species which appears on open, bare depression wetlands at the start of the growing season, and increases in cover until it produces fruiting bodies at the end of the wet period. It is a habitat indicator species preferring sandy clay soils, occurring with the low, spreading forb, *Alternanthera nodiflora* in Community 2, 3, 6 and Sub-Community 1.3 and forms associations with other members of Species Group I (S1 Appendix), *Schoenoplectus muricinux*, and *Eragrostis rigidor*, a medium-tall grass, also preferring sandy clay soils. *Marisela capensis* has a looser ecological association with the low, creeping grass, *Cynodon dactylon* and the taller, semi-lax *Agrostis lachnantha*, both OBL species. *Marisela capensis* was found at sites such as De Dam (S2 Appendix, p006bftddmm), Dealsville (S2 Appendix, p009deaqwgg) and Seven Dams (S2 Appendix, p014blo7dms).

### Wetland-types

**Fig 5:**
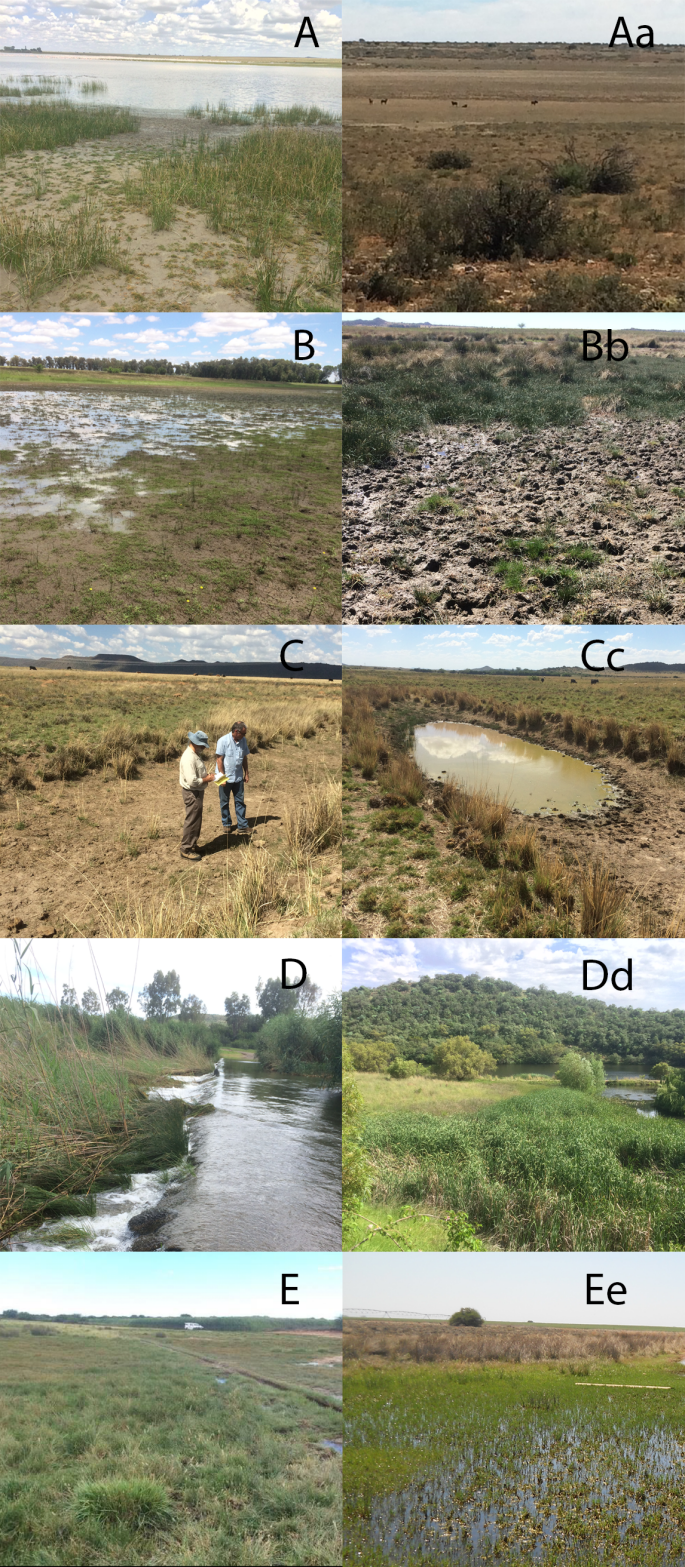
**Categorization of five, freshwater wetland depressions-types with descriptions of vegetation and ecology.** A. Deelpan, a typical saline endorheic pan, with narrow, the dense vegetated pan-margin, providing ideal breeding habitat for *Aedes*. Aa. Holpan, a non-saline pan, covered with *Eragrostis bicolor* the low, caespitose, specialist arid-region grass. B. De Dam, shallow depression wetland with clay soil and emerging sedge *Fuirena coerulescens*, grass *Echinochloa colona* and fern, *Marisela capensis*. Bb. Petrusburg wetland with large-tufted *Scirpoides dioecious*, emerging *Cyperus laevigatus* sedges, and the spreading, prostrate forb *Hypertelis salsoloides*. C. Mature ox-bow cut-off, 100cm deep, with wetland vegetation, sedges, grasses, on the margins. Cc. Inundated ox-bow wetland-type at Bougainvillea, a site of high sheep mortality during the 2010 outbreak. D. Riet River in flood, near Mokala National Park, with dense, monotypic stands of *Phragmites australis*. Dd. Seven Dams had no RVF mortalities. The most species-rich wetlands with extensive stands of *Phragmites australis* (foreground) and *Typha capensis*. E. Sedge and Juncus dominated wetland. The deep grove is created by the wheel of the pivot irrigator. Ee, Extensive, spill-over wetland created at Rooibokpan near Jacobsdal, dominated sedges, *Juncus* and OBL forbs in the <50 cm deep water.

Classification of pans and the associated wetland vegetation in the western Free State has previously been undertaken, covering an area of 41,819 km^2^, with 8,803 salt pans counted for this region [25]. Four pan-types were described based on vegetation structure and the presence of emergent vegetation: 1. Bare and scrub pans 2. Sedge pans 3. Mixed grass pans 4. *Diplachne* (Poaceae) pans. More recent and detailed work of Collins [53] and Mucina and Rutherford [24] adds to Geldenhuys’ classifications, and also leaves out the anthropogenic wetlands. This study categorises five wetland types (Fig 5 and S5 Appendix), included an additional classification of anthropogenic wetlands, as well as the previously described palustrine, freshwater wetlands at Seven Dams Conservancy in Bloemfontein [54], that are not pans or playas.

Five categories of wetlands were identified; 1. Endorheic salt pans, 2. Non-saline depressions, 3. Palustrine wetlands, 4. Riparian wetlands, 5. Anthropogenic Wetlands. They are illustrated in Fig 5, A to E, presented in Table 1 (linking mosquitos collected and vegetation), and fully described in S5 Appendix; ‘Categorization of five, freshwater wetland depressions-types with descriptions of vegetation and ecology’.

**Table 1:** Five wetland category types, location, the association with mosquitoes, dominant vegetation, geology, soils and site identification number.

Site: Farm name, nearest town.	Cate-gory	Wetland type	Dominant vegetation. Mosquitos (Msq) collected adult(a),pupae (p),larvae (l).	Geology, soils, notes	Farm identification number
Graspan/ Holpan/ SANPark, Kimberley	1	Depressions, endorheic non-saline pans.	grass/invasive plants. No OBL species. Msq; *Aedes*, a.	Andesite lava & modern, red Kalahari aeolian soils	p015kimgrsp
Deelpan (Flamingo Pan), Brandfort.	1 & 2	Upland depressions, possibly palaeo-river.	Sedges/*Juncus*. Msq; a,l,p.	Endorheic salt pan. Calcrete outcrops. Ecca, Beaufort shales.	p013bradlpn
Lamarloo, Bultfontein.	1 & 2	Permanent wetland embedded in salt pan.	Sedge/*Juncus*. Msq; a, l, p.	Calcrete. Endorheic salt pan.	p004bullmrl
Martinusrus, Petrusburg.	1 & 2	Shallow depression, open salt pan.	Sedges/*Juncus*. Msq; a.	Saline pan, Calcrete. Ecca, Beaufort shales.	p011petmrtn
Rietpan, Soetdoring.	1 & 2	Open pan, margin and inflow channel.	Sedges/*Phragmites* None, discontinued site.	Calcrete outcrops. Ecca, Beaufort shales.	Discontinued
Witkraal, Brandfort	2	Upland depressions, possibly palaeo-river.	Sedges/grass. Msq; a.	Ecca, Beaufort shales	p001brawtkr
Weltevrede, Bultfontein.	2	Depression, palaeo-rivers, seasonally inundated.	Grass/sedge. *Juncus*? *Aedes*; a.	Karoo sediments, Ecca, Beaufort group grey shales.	p002bulwltv
Adamshoop, Oppermansgronde	2 & 3	Palustrine wetlands. Depressions.	Sedges/grass Msq; a.	Clay soils from Ecca, Beaufort Sandstone	p008oppdmsh
Brakput, Koffiefontein.	2 & 3	Palustrine wetlands. Depressions.	*Juncus/Phragmites/* Sedges. Msq; a.	Calcrete, Hornfels with dolerite fragments.	p005petbrkp
De Dam, De Brug.	2 & 3	Depression, palaeo-rivers, and palustrine wetland.	Sedges/grass. Msq; a.	Calcrete, shale, red aeolian sandy soil. Gray Beaufort shales.	p006bftddmm
Mooigekry, Bultfontein.	3	Palustrine seasonally inundated.	Sedge. No mosquitoes	Karoo Super-group. Ecca, Beaufort Sandstones, grey shale.	p003bulmgkr
Quaggafontein, Dealesville.	3	Artesian spring-fed wetlands.	Sedges/*Juncus* Msq; a,l,p.	Calcrete 3–4 m thick. Ecca, Beaufort shales.	p009deaqwgg
Seven Dams, Bloemfontein	3	Palustrine wetlands.	Sedges. Msq; a.	Extensive Dolerite sills.	p014blo7dms
Bougainvillea, Reddersburg	3 & 4	Palustrine wetlands. Riverine wetlands.	Sedge/grass/*Juncus* Msq; a,l,p.	Clay soils from Ecca, Beaufort Sandstone	p012redbgnv
Riet River, Mokala National Park	4	Riverine, permanent river.	Sedge/grass/reeds Msq; a.	Andesite lava. Alluvial soils	16C-002kimmkln
Poortjiesdam Farm, Oppermansgronde.	5	Anthropogenic dam, shallow depression	Weeds, sedge/*Juncus*. Msq; *Culex*; l,p, in crib.	Dolerite hills and Ecca, Beaufort shales	Discontinued site
Rooibokpan Agricultural research station, Jacobsdal	5	Anthropogenic floodplain, irrigation dam overflow.	Sedges/grass. Msq; *Culex*; a, moderate numbers	White, salt-leached soils. Ecca, Beaufort shales	p010jacrtrv
Waterput, Luckhoff.	5	Anthropogenic wetland made by pivot irrigating.	Sedge/grass. Msq; a,l,p.	Endorheic saline pan & upland depression. Ecca, Beaufort shales	p007lucwtrp

Fig 5A (saline) and 5Aa (non-saline). Show category one, endorheic pans. The distinct vegetation difference from the pan to the uplands is the result of hyaline, anaerobic soils, and the vegetation response to these conditions. Fig 5B and 5Bb. Show category 2, non-saline depressions. Fig 5C and 5Cc. Illustrate category 3 palustrine wetlands. Fig 5D and 5Dd. Illustrate category 4 riparian wetlands. Fig 5E and 5Ee. Illustrate category 5 anthropogenic wetlands. Highly saline soils have been created by the constant watering.

The five-wetland-categorization was derived from field observations, and classified according to principles based on geology, soil colour and texture, vegetation-type, and physiognomy and presence/absence of surface water. The compilation in Table 1 shows these five categories with location of mosquito’s collected and the unique farm-sites identification number. This categorization includes a new, previously undescribed category of ‘Anthropogenic Wetlands’. Components of this categorization form part of the existing azonal wetlands [24]; however, in South Africa the anthropogenic component has not previously been included in phytosociological investigations and descriptions.

Upland depressions fall within three categories: category 1; endorheic, non-saline pans–largely found at Graspan/Holpan National Park, category 2; shallow, freshwater wetlands embedded in salt pans, and category 3; the most extensive, shallow wetlands, which are possibly the results of the Palaeo-Kimberley River [27] and have included sites for collection of large numbers of *Aedes* over the last 20 years (Kemp pers. Comm.). The category 3 wetlands (Table 1) also coincide with the regions of high mortality during the 2010 outbreak [8].

### Phytosociological analysis

The phytosociological analysis clusters the wetland vegetation into 8 communities, 7 sub-communities and two variants (Table 2 and S1 Appendix). Overlap exists with dominant and diagnostic plant species clustered into communities and sub-communities; with the names designated for each cluster (Community or Sub-community) given as the highest order unit. Overlap also exits with some individual species or species groups; however, as their occurrence is either of low order, ‘+’ or ‘r’, or scattered over relevés, these species are not used in the formal nomenclature, but may be used in Results or Discussion. The structure and species composition of vegetation communities is not absolute; it will change over time and will also depend on the observer’s view point. A degree of stochasticity is always inherent in any and all systems [55], whether for theoretical considerations, analysis and interpretation, or for wetland vegetation composition and structure.

**Table 2:** Relationship between named plant communities, vegetation characteristics and reported RVF mortalities.

Community	Name	Defining Species	Indicator Species	Dominant Species	RVF mortality reported
Community 1	*Eragrostis bicolor*, semi-arid grassland	*Eragrostis bicolor*	None	*Eragrostis bicolor*	None
Sub-community 1.1	*Cynodon dactylon – Tragus berteronianus*, semi-arid grassland	*Cynodon dactylon*	None	*Tragus berteronianus*	None
Sub-community 1.2	*Geigeria filifolia – Eragrostis bicolor*, semi-arid grassland	*Geigeria filifolia*	None	*Eragrostis bicolor*	None
Sub-community 1.3	*Falkia oblonga—Urochloa panicoides*, semi-arid grassland	*Falkia oblonga*	None	*Urochloa panicoides*	None
Sub-community 1.4	*Eragrostis bicolor*, semi-arid grassland	*Eragrostis bicolor*	*Albuca virens*, *Dipcadi viride*, arid regions, sandy, well-drained soils.	None	None
Sub-community 1.5	*Stachys hyssopoides – Eragrostis bicolor*, semi-arid grassland	*Stachys hyssopoides*	None	*Eragrostis bicolor*	None
Variant 1.5.1	*Eragrostis bicolor*, semi-arid grassland	*Eragrostis bicolor*	None	None	None
Variant 1.5.2	*Cynodon dactylon*, grassland	*Cynodon dactylon*	*Denekia capensis*, *Lobelia angolensis*, *Crassula natans*.	None	None
Community 2	*Cyperus laevigatus – Agrostis lachnantha*, sedge/grass wetland	*Cyperus laevigatus*	*Isolepis cernua*	*Agrostis lachnantha*	High 90–180
Community 3	*Fuirena coerulescens – Echinochloa colona*, sedge/grass wetland	*Fuirena coerulescens*	*Moraea polystachya*, *Albuca prasina*	*Echinochloa colona*	High 90–180
Community 4	*Hemarthria altissima – Schoenoplectus muricinux*, grass/sedge wetland	*Hemarthria altissima*	*Sebaea pentandra*, bare sandy soils	*Schoenoplectus muricinux*	High 90–180
Community 5	*Cyperus laevigatus – Pseudschoenus inanis*, sedge wetland	*Cyperus laevigatus*	*Lagarosiphon major*, *Crassula natans*, *Eleocharis dregeana*, *Limosella grandiflora*	*Pseudschoenus inanis*	High 90–180
Community 6	*Agrostis lachnantha – Cyperus longus*, grass/sedge wetland	*Agrostis lachnantha*	*Pentzia globose*, *Hordeum stenostachys*,	*Cyperus longus*	None
Community 7	*Scirpoides dioecious – Juncus rigidus*, sedge/Juncus wetland	*Scirpoides dioecious*	*Leptochloa fusca* erect and creeping forms.	*Juncus rigidus*	Highest 180–450
Community 8	*Cyperus laevigatus – Juncus rigidus*, sedge/Juncus wetland	*Cyperus laevigatus*	None	*Juncus rigidus*	Highest 180–450
Sub-Community 8.1	*Cyperus marginatus – Schoenoplectus triqueter*, sedge wetland	*Cyperus marginatus*	*Schoenoplectus triqueter*, *Rorippa nasturtium-aquaticum*	*Schoenoplectus triqueter*	High 90–180
Sub-Community 8.2	*Scirpoides dioecious – Hypertelis salsoloides*, sedge/succulent forb wetland	*Scirpoides dioecious*	None	*Hypertelis salsoloides*	High to very high 80–450

### Descriptions of plant communities, ecological parameters and associated RVF mortalities

All plant communities are presented in the synoptic table S1 Appendix. The relationship between plant communities, vegetation and numbers of RVF mortalities are compiled in Table 2. Numbers of RVF mortality show in the extreme right column, are derived from the RAS, OIE Report 17 [8]. They are fully named and described in detail in S5 Appendix.

Community 1 is defined by the single grass species *Eragrostis bicolor*, and with *E*. *obtusa* are both species indicative of adaptation to arid areas. No *Juncus* or *Typha* were found with almost a complete absence of all sedges (Table 2 and S5 Appendix). No RFV livestock mortalities where recorded from this area, but adult *Aedes* were caught on one plot at this site.

Community 2 is dominated by the sedge *Cyperus laevigatus* and the Obligate Wetland bunch-grass *Agrostis lachnantha* indicate wetlands capable of holding water for several months. The associated halophytic succulent shrubs (*Salsola kali*, *S*. *glabrescens*) indicate wetland community on the margins of endorheic salt pans (Table 2 and S5 Appendix).

Community 3 is dominated by the low sedge *Fuirena coerulescens* and the low, spreading grass *Echinochloa colona*, species indicative of increased habitat wetness (Table 2 and S5 Appendix). Community 3 vegetation include sites which recorded some of the highest Rift Valley fever livestock mortality rates during the 2010–11 outbreak.

Community 4 is dominated by the tall, mat-forming, grass *Hemarthria altissima* and the sedge *Schoenoplectus muricinux* and form the second most species-poor communities (Table 2 and S5 Appendix).

Community 5 comprises two sedges, *Cyperus laevigatus* and *Pseudschoenus inanis*, and is the wettest of all the associations (Table 2 and S5 Appendix), found as upland depressions in areas of reported high RFV livestock mortality. *Culex* mosquitoes where found but no *Aedes*.

Community 6 comprises the grass *Agrostis lachnantha*, and the sedge *Cyperus longus*, and is the most species rich of all the associations (Table 2 and S5 Appendix). These are palustrine wetlands, and occur on the dolerite found at 7 Dams Conservancy, Bloemfontein. No RVF mortalities were reported during the 2010 outbreak.

Community 7 is dominated by the tall sedge, *Scirpoides dioecious* and rush *Juncus rigidus*, a habitat specialist for sandy, hyaline soils (Table 2 and S5 Appendix). The wetland vegetation forms associations found at open depressions. Moderate to high mortality rates were recorded from these sites during the 2010–11 outbreak.

Community 8 is the largest of all the communities with two sub-communities. It is dominated by the sedges *Cyperus laevigatus*, *C*. *marginatus*, *Schoenoplectus triqueter*, and the rush *Juncus rigidus* (Table 2 and S5 Appendix). Sub-community 8.1 has a significant organic component found on the margins of endorheic salt pans such as Lamarloo (S2 Appendix, p004bullmrl) near Bultfontein, sites recording some of the highest reports of livestock mortalities during the 2010–11 outbreak. Sub-Community 8.2 comprises open, shallow wetland depressions and the anthropogenic wetlands at Jacobsdal (S2 Appendix, p010jacrtrv) and Luckhoff (S2 Appendix, p007lucwtrp). Medium to low mortality rates were recorded during the outbreak. Limited numbers of *Culex* adults and larvae were collected but no *Aedes* were detected at the time of the phytosociological survey.

Table 3 compiles all the livestock data presented in detail by farm for the 2010 outbreak. It clearly shows sheep susceptibility (265 080), confirmed cases (13 117) and deaths (8 078) to be an order of magnitude greater than all other livestock and wildlife deaths combined (susceptible 91 318; cases 1 225; deaths 799).

**Table 3:** Animal cases and deaths during the 2010 Rift Valley fever outbreak.

Species	Susceptible	Cases	Deaths	Destroyed
Sheep	265 080	13 117	8 078	512
Cattle	70 445	738	448	7
Goats	5 993	157	86	11
Goats/sheep	5 163	269	204	1
Wild species	9 344	52	52	0
Camelidae	227	5	5	0
Buffaloes	146	4	4	0
**Totals**	**356 398**	**14 342**	**8 877**	**530**

Total outbreaks = 489 farms. Data derived from RSA, OIE Report 17, pp. 98.

Table 3 also links ‘Source of the outbreak(s) or origin of infection’ as ‘Vectors’, and reported cases and deaths due to RVF with farms. The field-study sites were selected from farms in RSA, OIE report 17 [8] which recorded high numbers of deaths. This enables the study to link the vegetation ecology to high animal mortality in the 2010 outbreak (Fig 2 and S5 Appendix).

## Discussion

Of the 8 communities which make up the vegetation of the entire study area, there is a distinct difference between Community 1 and the other 7 communities. Species common to most communities include the most widely occurring *Cynodon dactylon*, a low (10-15cm), mat-forming grass, also found in the variant 1.5.1 (S1 Appendix; Materials and Methods, Naming the plant communities) where it occurs as the dominant species. Other species found only in Communities 2–8 include the OBL graminoids, *Agrostis lachnantha*, the sedges, *Cyperus laevigatus*, *C*. *longus*, *C*. *marginatus*, and the thin forb *Pseudognaphalium luteo-album*. The tall, densely tufted, sharp-tipped sedge, *Scirpoides dioecious* grows in half the sites, with the medium-tall rush, *Juncus rigidus* growing in Communities 4, 6, 7 and 8. The members of species group Z (S2 Appendix), are all OBL or FACW species and form a weak, scattered but discernible association thinly-spread over Communities 2 to 8.

Wetland vegetation is considered to be azonal [24]. However, it is embedded in the vegetation matrix of the surrounding biome. Most of the vegetation of the study area falls within the Grassland Biome [24] which would correspond with the dambo located in the ‘bushed grasslands’ described by Linthicum [9], as ‘Ecological zone II; p. 228’. The emergent vegetation at the dambo site is primarily *Cyperus immensus* while the tall grass, *Digitaria abyssicina*, predominated in the rest of the temporary dambo—wetland. For the Free State, South Africa, the Savanna and Nama Karoo Biomes comprise the remainder of the vegetation which occurs in the Graspan/Holpan National Park, and the anthropogenic wetlands in the southwestern Free State.

Three distinct ecological zones are reported by Arum et al. [23] for their study in the North east region of Kenya. These zones are semi-arid, dry humid forest and humid to dry sub-humid. In the Lamu, Garissa regions of Kenya, mosquito genera know to be vectors for RVFV [56], show a preference for resting sites on certain plant species [23], with the suggestion that knowing which plant species are preferred by these two genera, may help with identifying breeding sites for RVFV mosquito vectors. The vegetation and the species composition are wetland taxa, and, because of the harsh redox conditions in wetlands, will remain relatively unchanged over time, despite short-term increase and decrease of rainfall. Mosquitoes also feed on the plant sap as a source of sugars [23], and such plant communities may be some distance away from wetlands as with the Bultfontein site where the farmer has reported swarms of mosquitoes rising from alfalfa fields adjacent to his farm house (Mr. Kobus Steenkamp, pers. comm., 2014). The space under the leaves and tightly-packed stems, provide a habitat with lower ambient temperature and increased humidity. They postulate that these cool, moist areas are ideal for *Aedes* and *Culex* and also provide plant-sap as food [23].

### Vectors and flight distance

**Fig 6:**
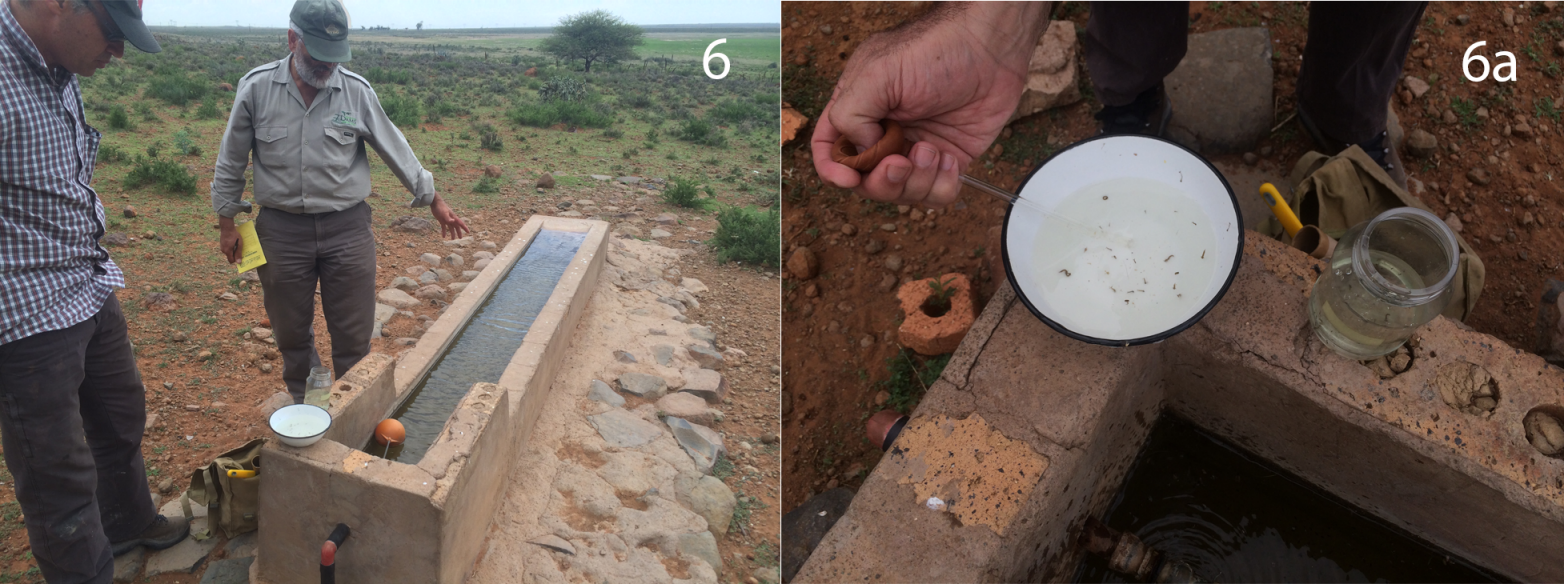
***Culex* pupae and larvae (known RVF virus amplifying-vectors), found at Oppermansgronde.** Sheep trough full of Culex, capable of 2 km flight from wetlands, a known amplifying *Aedes* RVF vector. a. Culex pupae and larvae, more than 400m from any pan or wetlands, at a confirmed high mortality site during the 2010 RFV outbreak.

Adult *Aedes* and *Culex* were captured during this study. Sites included a water-trough in Oppermansgronde (Table 1, p008oppdmsh), used by sheep, in which dozens of *Culex theileri* sp?, larvae and pupae were found (Fig 6 and 6A), and adult *Aedes* sp?, in a small, 7 m diameter wetland, designated Buffalo Pan in the Graspan/Holpan National Park (Table 1, p015kimgrsp).

**Fig 7:**
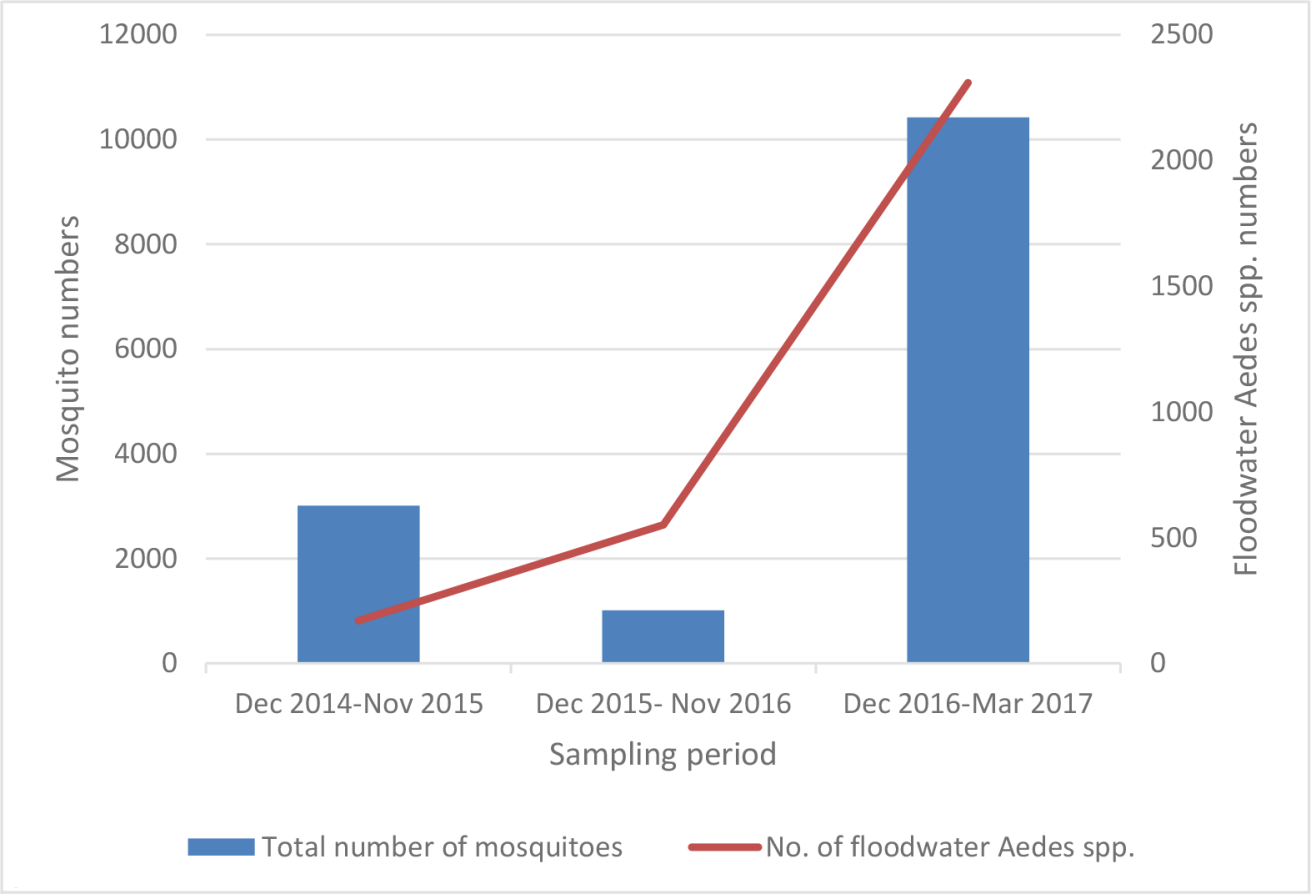
Annual comparison over 3 years of numbers of *Culex* and *Aedes* collected. The columns show the clear relationship between rainfall and mosquito numbers. The annual comparison of mosquito samples from 2014 to 2017 is derived from the Rift Valley fever virus vector surveillance work package.

The analysis of vector sampling was not an aim of this paper. However, collection of mosquitoes was done simultaneously with the vegetation work, during 2014 and 2015 but, due to the drought conditions during 2015 and 2016, conditions for mosquito breeding was not ideal and very few mosquitos where collected. Rainfall for 2016 and 2017 has improved with over 13 900 adult mosquitoes collected, of which 5542 have been identified, with 5226 tested but with none found to be infected with RVF virus [57]. Fig 7 shows the affects rainfall and a consequent reduction of habitat, on total numbers of mosquito collected over three rainy seasons, from 2014 to 2017. Additionally, when the study is completed in 2019, a full analysis of 5 years of vector sampling and distribution starting from 2014 will be done and will be the subject of a different paper.

Floodwater *Aedes* are local and the *Culex* are the dispersing agents; mosquito dispersal via active flight is about 300 metres for *Aedes mcintoshi* and at least 2 km for *Culex theileri* (pers. Obvs., Kemp), [58]. However, the access to plant nectars/high-sugar-content sap can extend mosquito flight ranges considerably. Additionally, thunder storms involve strong vertical and horizontal winds which are logically capable of dispersing mosquitoes over much greater distances [59]. There is no evidence to support this in South Africa, but there are many hypotheses and examples of insect dispersal elsewhere [60, 61, 62].

### Survival of mosquito eggs

**Fig 8:**
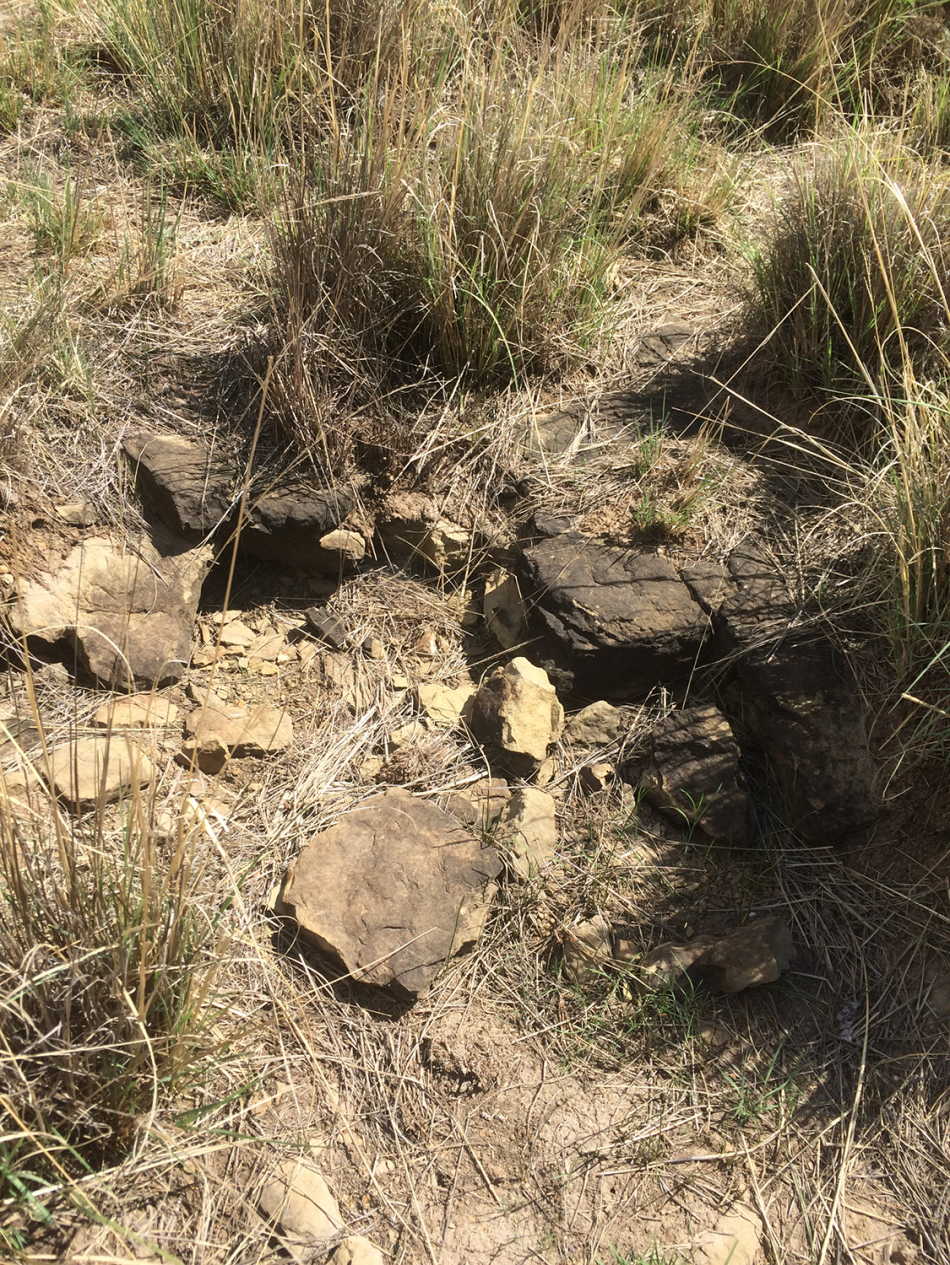
Palustrine wetland vegetation matrix, clay soils and sandstone habitat for *Aedes*. Wetland vegetation matrix of sedges; *Scirpoides dioecious*, *Cyperus laevigatus*, and the grass *Miscanthus junceus*, on high clay-content soils from Ecca series sandstone and shales found at most sites.

Spot temperatures taken during the survey ranged from 18°C in the rain, taken on grass tufts, at 10.30 am, to the highest, in-sun temperatures of 71°C, on dead vegetation-matrix, at 11.35 am. The average was 42°C, on substrates varying from bare, grey soil, live grass tufts, vegetation matrix and mud (Fig 8).

The vegetation and ecological study found 9 wetland plant associations characterised by the presence of hydrophilic grasses, sedges, rushes and forbs. The most species-rich communities occurred on the dolerite soils found in Bloemfontein which also receives the highest rainfall of all study sites. The wetland vegetation found on saline-soils underlain by shales producing high-clay content soils constitutes the majority of study sites and is coincident with the centre of the RVF 2010–2011 outbreak. The vegetation is dominated by OBL or FACW plants including; *Juncus effusus*, the sedges *Cyperus laevigatus*, *C*. *marginatus*, *Scirpoides dioecious* (all FACW), and the grasses, *Agrostis lachnantha* (OBL), *Cynodon dactylon*, *C*. *transvaalensis*, *Sporobolus albicans* (all FACW), *Phragmites australis* (OBL), *Paspalum distichum* (FACW), a wide-spread alien wetland grass species, and *Eragrostis bicolor* in the more arid areas. Significant wetland forbs include *Pseudognaphalium luteo-album*, *Ranunculus multifidus*, *Rumex lanceolatus* (all 3 FACW), and *Veronica anagallis-aquatica* (OBL).

Surprisingly few alien, invasive species where recorded, and include, *Bidens bipinnata*, *B*. *pilosa*, *Cirsium vulgare*, *Cosmos formosa*, *Oenothera rosa*, *Panicum coloratum*, *Paspalum distichum*, *Schkuhria pinnata*, *Tageties minuta*, *Verbena bonariensis* (all FACW) and *Veronica anagallis-aquatica*.

An overlay of the geological map showing the South African Karoo Supergroup with the outbreak/wetland sites shows a striking correlation between the shales of the Ecca and Beaufort series, and the clay-rich, water-retaining soils produced from shale, the wetland types and the subsequent wetland vegetation. All these factors come together in the west/central Free State which has a plethora of these endorheic pans and Palaeo-Kimberley river wetlands which may provide appropriate habitat for RVFV mosquito vectors [63]. The highest mortality during the 2010 RVF outbreak occurred in the Free State where all these factors combine. Karoo Supergroup sediments also extend into Tanzania and Kenya from the Lamu Basin where RVF is endemic [64, 65].

The other major environmental factor necessary for a RVF outbreak is sufficient, heavy rainfall to provide for the completion of the aquatic stages of the *Aedes* life-cycle (Alan Kemp, pers. comm.). Markedly lower rates or zero livestock deaths occurred where the geology is not Karoo Supergroup sediments. Graspan/Holpan sites are on Andesite lava which erodes to a high iron content, red soil with little clay. *Aedes* were found at one of the small wetlands but in very low numbers, which may or may not be indicative of the suitability of these soils but could also be as a result of the limited rainfall at the time of the study.

Flooding of the breeding habitat of RVFV-competent *Aedes* spp. vectors is most likely the single, major cause driving the emergence of RVFV. The two principal vectors in our study area are *Aedes mcintoshi* and *Culex theileriAedes mcintoshi* acts as primary or maintenance vector, capable of maintaining RVFV transovarially through desiccation-resistant embryonated eggs, and *Culex theleri* acts as secondary, amplifying vector [58]. Primary vectors tend to remain in the immediate vicinity of their natural pan breeding sites and initiate localised, rarely detected transmission cycles. In contrast, the *Culex* vectors disperse widely to utilize anthropogenic wetlands and to feed on vertebrate hosts, leading to greatly enlarged mosquito populations. As a result, these *Culex* species are capable of extensive dispersal of RVF virus and amplification of the RVF outbreak [9, 63]. This likely explains why localised heavy rainfall does not precipitate an epidemic.

The wetland vegetation and associated environmental parameters show the following gradients:

1. The Graspan/Holpan area to the west in the Northern Cape, is the driest; precipitation < 250mm. The geology is Andesite, producing low-clay soils with limited water retention properties. The vegetation is dominated by the low grass *Eragrostis bicolor* with a few sedges and limited *Juncus* spp. The region has low records of RFV mortality in livestock, though *Aedes* mosquitoes have been found at Buffalo pan.
2. The main study area; Reddersburg in the south, Bultfontein in the north with Brandfort, Dealsville, Petrusburg, Koffiefontein and Oppermansgronde centrally has moderate to high precipitation from 450mm-350mm. The geology is Karoo-Supergroup sediments, Beaufort sandstones with the grey shale horizon present throughout most of the site, which produces high clay-content soils. It is this central region of the Free State which has most of the endorheic salt pans, upland depressions resulting from the Palaeo-Kimberley River, and palustrine wetlands. It is also the region of highest recorded mortality for the 2010 outbreak. The vegetation is a component of the country-wide, azonal freshwater wetlands and is dominated by OBL and FACW wetland species which include sedges, grasses and rushes. It provides the ideal habitat for *Aedes*. During this study, floodwater *Aedes* were collected at Buffalo pan, and in previous years at Mooigekry and Weltevrede (Alan Kemp, pers. comm., 2016), [63, 64].

Continued monitoring of the vegetation should continue using the Braun-Blanquet method for 2 reasons:

1. to document the change in cover and,
2. to document the succession of plant species resulting from the change in climate due to the Walker Circulation, which initiated the development of La Niña conditions and a positive Southern Oscillation Index in late 2016. A better understanding of salinity, pH, water temperature and dissolved oxygen content is needed. Future studies should concentrate on acquiring more data to establish daily and seasonal variations, describe associations and examine for periodicity and patterns possibly responsible for *Aedes* eggs surviving in wetland soils and vegetation. Understanding the complex link between rainfall, geology, soil structure, wetland type and the associated vegetation may provide information for management of floodwater *Aedes* spp. Populations in order to mitigate against catastrophic RFV outbreaks.

## Conclusion

In South Africa, high Rift Valley Fever mortality in the 2010 outbreak was concentrated in the region where numerous upland depression wetlands occurred. The geology of Communities 2, 3, 4, 5, 7 and 8 are all part of Ecca and Beaufort sandstone and shale which weather to produce high clay-content soils. These wetlands are also aligned with the Palaeo-Kimberley River system.

This paper identified important characteristics of wetlands from areas where known RVFV mortalities have occurred (and that presumably were initiated by the mosquito vectors that use that habitat). All sites with RVF mortality had vegetation consisting of sedges, *Juncus* and grasses, all of which are Obligate or Facultative Wetland species. It is known that the primary RVF *Aedes* vectors (that sustains transovarial transmission of RVFV) breed in these areas and that, when flooded, the wetland would support the amplifying vectors of the *Culex* genus. Wetland vegetation provides the appropriate and (likely) ideal habitat for the floodwater species to lay eggs, hatch, and rest under the cool conditions provided by the vegetation while waiting to feed on and spread the virus to livestock that use the wetlands as watering points and for forage.

The vegetation and associated factors (soil moisture, cool temperatures) found in community 2 to 8 wetlands, are likely condition for the emergence and propagation of RFV vectors. Of the 129 sample sites, Community 1 consisted of 27 relevés and constituted 21% of the total vegetation sampled. No records of RVF mortality were recorded from any of these sites. The vegetation contains almost no Obligate or Facultative Wetland species and is defined rather by species adapted to arid conditions in low-lying areas in which water accumulates after rain. The hydrogeomorphic conditions are distinctly different from those of the remaining 102 sample sites which have the common graminoid wetland vegetation.

Of the remaining sites, the most dense wetland vegetation was found in communities 7 and 8 and comprise 49 relevés which constitute 38% of the total vegetation sampled. Communities 7 and 8 share the dominant and diagnostic species of *Juncus rigidus*, *Scirpoides dioecus*, *Sporobolus albicans* and *Cyperus laevigatus* with communities 2 (12 relevés) and 5 (8 relevés), along with *Cyperus longus*, which is also a dominant and diagnostic species for community 6 (17 relevés). Communities 2, 5, and 6 constitute an additional 28.7% of the vegetation. All these species are Obligate or Facultative Wetland species and the 37 relevés from communities 2, 5, and 6 combined with the 49 from communities 7 and 8 comprise 66.7% of the total vegetation. The remaining vegetation found in community 4 (11 relevés) have anaerobic, hydrogeomorphic conditions suitable for Obligate and Facultative Wetland species, despite not having extensive or dense wetland vegetation such as found in communities 2, 5, 6, 7 and 8. All of the vegetation from communities 2, 5, 6, 7, 8 and the remaining from community 3 (11 relevés), and community 4 (3 relevés), had records of RVF mortality and combined, constitute 77.6% of the vegetation sampled at all the sites. Of all 129 sites sampled (minus two left out of the formal classification), 77.6% of all the vegetation, have documented cases of mortality due to RFV.

The wetlands and pans selected at each farm/park represent, phytosociologically, the overall vegetation found on the whole farm. And it is these pans and wetlands which are known breeding sites suitable for Aedes as primary vectors and which have secondary breeding sites for Culex, the amplifying vector for RFV virus.

Understanding the complex link between wetland vegetation, rainfall, geology, soils, and palustrine or wetland depression, may provide information for management of floodwater *Aedes* mosquitoes by identifying the most suitable mosquito breeding sites which could then be targeted to implement appropriate vector-control strategies to mitigate against catastrophic RFV outbreaks.

## Supporting information

S1 Appendix Synoptic table for Rift Valley Fever wetland study, Free State, South Africa. (XLSX)

S2 Appendix A list of all plants collected, author’s names and voucher numbers. (XLSX)

S3 Appendix VegCap Rift Valley Fever Study raw Braun-Blanquet data. (XLSX)

S4 Appendix RVF Site ID’s. (XLSM)

S5 Appendix Geology, Soil and Land types, Categorization of five freshwater wetland depression-types with descriptions of vegetation and ecology Syntaxonomical description of plant communities and analysis of the ecological parameters. (DOCX)
