# Supplementary material for: A phytosociological analysis and description of wetland vegetation and ecological factors associated with locations of high mortality for the 2010-11 Rift Valley fever outbreak in South Africa
Source: PLoS One. 2018 Feb 20;13(2):e0191585. doi: 10.1371/journal.pone.0191585 (PMC5819772; doi:10.1371/journal.pone.0191585)
Supplement: S5 Appendix — (DOCX) [file pone.0191585.s005.docx]

# S5 Appendix. Supporting Information on geology, soils, formal plant community names and ecological description for: A phytosociological analysis and description of wetland vegetation and ecological factors associated with locations of high mortality for the 2010-11 Rift Valley fever outbreak in South Africa.

Robert F. Brand, Melinda K. Rostal, Alan Kemp, Assaf Anyamba,

Herman Zwiegers, Cornelius W. Van Huyssteen, William B. Karesh,

Janusz T. Paweska

# Geology.

The geology of the study area consists of two distinctly different rock-types: Karoo Supergroup sediments of sandstone and shales and the Ventersdorp Supergroup rocks of red, iron-rich, amygdaloidal andesite lavas. This comprises a large portion of the interior of South Africa. Dolerite is an igneous, black, fine-grained, saturated, crystalline rock which forms the major geological component of the 7 Dams Conservancy and completely overlays and intersects the blue-grey, Ecca shales found north of Bloemfontein [1, 2, 54].

Throughout the Free State, the study sites are underlain by the younger Ecca-group shales and the lower, Beaufort shales and sandstones. These two horizons are part of the Dwyka (Permian) to Ecca (early Permian) sediments; part of a huge, deep inland Karoo basin, a depositional site emplaced circa 300 to 65 million years ago [1, 2]. The topmost series of the Karoo Supergroup consists of Stormberg amygdaloidal basalt, a massive emplacement of fissure lavas up to 1000 m in depth, laid down over a two million year episode, at the end of the Gondwana split some 65 My years ago [2]. Since this orogeny, the major geological process of South Africa has been one of weathering and erosion occurring in the interior of the continent forming the current African Land-surface [24]. It is this lengthy erosional process that has produced the mature, flat, meandering palustrine rivers. The endorheic pans and upland depressions are the remains of a tectonically disrupted palaeo-river system [26, 27], which occurs throughout study area. These wetland systems were the sites of highest mortality during the 2010 outbreak, and of most interest for the study (Fig 2).

The far western part of the Free State and the study sites in the Northern Cape are composed of rhyolite lavas forming the ridges and aeolian deposits of Tertiary to recent ages accumulating in the shallow depressions. Calcrete, soft, easily weathered and white shales of the Tierberg and Whitehall formations are also present [3, 27, 25]. The large, shallow endorheic pans (Holpan and Graspan) are situated in this flat landscape with low clay-content soils.

# Soils and Land-types.

The major permanent river is the Riet River, which drains west into the Orange/Hearts River complex. Altitudes vary with Bloemfontein in the east the highest at 1 450 m with a gradual decline to Holpan/Graspan with altitudes of 1 050 m to 1 187 m [4, 24].

For the area situated on the Karoo Supergroup sediments, the land types are: Ae 46, Ae 49, Fc 13, Da 1, Da 13 and Da 103 [5, 6]. Soils vary and include Katspruit, Montague, Oakleaf and Rensburg soil forms [6], (Soil Classification Working Group, 1991). The soils may be sandy and are mostly clay-rich with some lime present. For the drier Kimberley region of Graspan/Holpan situated on andesite, the soil forms are: Ae44 or Fb1 with lime present and showing a carbonate horizon [5]. Soils have a demonstrable difference from the Karoo Supergroup sediments and include Addo, Augrabies, with Montague and Katspruit [5, 6, 24].

# Categorization of five wetland-types.

Five categories of wetlands where identified in the field. They are:

1. **Endorheic salt pans** (Figs 5A and 5Aa). These tend to be large pans with vegetation along the margins, may hold water for several years and are important sites for flamingos and waterfowl. A narrow band of halophytic sub-shrubs, sedges and rushes grow along the pan margin.

2. **Non-saline depressions** (Figs 5B and 5Bb). These may or may not contain wetland vegetation. These tend to be small, from several meters to 100 m in diameter, and may be circular or elongate in shape. It has been suggested that they may be the eroded remnants of palaeorivers [27]. The pans are seasonally inundated with grey, clay soils. At the start of the rains, the surface may be bare to sparsely vegetated. By the end of the growing season the pans may be completely covered by dense vegetation. The Holpan/Grasspan depressions have almost no OBL species, with deep red soils formed from andesite, not sandstones as with most of the other wetlands.

3. **Palustrine wetlands** (Figs 5C and 5Cc). These are meandering, ox-bow, mature stream-beds. They may be continuous or cut-off and isolated wetlands, narrow, elliptic or elongate, 1-20 m wide and a few to hundreds of meters long, and 0.5 to 5 m deep. They all have clay soils, with fully vegetated margins but may be vegetation-free in the centre, due to standing water for long periods after rain.

4. **Riparian wetlands** (Figs 5D and 5Dd). These are permanently flowing, rivers, or have flowing water for most of the year and are the most species-rich and ecologically diverse wetlands. The banks are lined with tall stands of *Phragmites* and *Typha* which form monotypic stands or are the dominant vegetation. The centre of the stream is generally open, flowing water. The Riet River and Seven Dams Conservancy are examples of these wetland-types.

5. **Anthropogenic Wetlands** (Figs 5E and 5Ee). These are created by irrigation and common to the western Free State and the result of pivot or flood irrigation supplied by canals from the Gariep Dam. The wetlands are either broad, open, floodplains or narrow, stream-like associations. The vegetation is dominated by sedge, *Juncus*, grasses with stands of *Phragmites*, as well as halophytic and succulent shrubs on the margins. They have created leached, high-salt content soils in areas which are historically arid and not suitable for agriculture. These anthropogenic wetlands are a new categorization as previously they have been left out of formal, phytosociological surveys.

# Syntaxonomic description of plant communities and analysis of ecological parameters.

All 8 plant communities, 7 Sub-communities and 2 variants are presented in the synoptic table, Appendix 1 and named as follows:

Community 1. *Eragrostis bicolor*, semi-arid grassland

Sub-community 1.1 *Cynodon dactylon*  *̶*  *Tragus berteronianus*, semi-arid grassland

Sub-community 1.2 *Geigeria filifolia ̶*   *Eragrostis bicolor*, semi-arid grassland

Sub-community 1.3 *Falkia oblonga - Urochloa panicoides*, semi-arid grassland

Sub-community 1.4 *Eragrostis bicolor*, semi-arid grassland

Sub-community 1.5 *Stachys hyssopoides ̶ Eragrostis bicolor*, semi-arid grassland

Variant 1.5.1 *Eragrostis bicolor*, semi-arid grassland

Variant 1.5.2 *Cynodon dactylon,* grassland

Community 2. *Cyperus laevigatus ̶ Agrostis lachnantha*, sedge/grass wetland

Community 3. *Fuirena coerulescens ̶ Echinochloa colona*, sedge/grass wetland

Community 4. *Hemarthria altissima ̶ Schoenoplectus muricinux*, grass/sedge wetland

Community 5. *Cyperus laevigatus ̶ Pseudschoenus* *inanis*, sedge wetland

Community 6. *Agrostis lachnantha ̶ Cyperus longus*, grass/sedge wetland

Community 7. *Scirpoides dioecious ̶ Juncus rigidus*, sedge/Juncus wetland

Community 8. *Cyperus laevigatus ̶ Juncus rigidus*, sedge/Juncus wetland

Sub-community 8.1 *Cyperus marginatus ̶ Schoenoplectus triqueter*, sedge wetland

Sub-community 8.2 *Scirpoides dioecious ̶ Hypertelis salsoloides*, sedge/succulent forb wetland

The plant community and associated ecological descriptions are presented in detail in S1 Appendix and Table 2.

## Ecological parameters.

Community 1 vegetation has a single sedge and very few wetland-indicator grasses or forbs. The community is mostly small, low-spreading arid-area plants, dominated by grasses, invasive species and spiny forbs. The vegetation of Community 1 is located on six pans on the Holpan/Grasspan South African National Parks Reserve (Table 1, p015kimgrsp), and is the furthest west of the study sites in the arid, Kimberley area. Previously, Holpan/Grasspan National Park (Table 1, p015kimgrsp) consisted of a number of severely overgrazed farms. The dense growth of the invasive species *Schkuhria pinnata* is evidence of this. No RVF mortalities where reported from Holpan/Graspan (Table 1, p015kimgrsp) or adjacent farms. The significant difference in vegetation between the Holpan/Grasspan (Table 1, p015kimgrsp) and the other sites to the east which recorded high mortality is the almost complete absence of OBL and FACW species of sedges, *Juncus*, grasses and forbs (S1 Appendix). Facultative, FACU and even some UPL species occur in the pans (S1 Appendix). For the entire Holpan/Grasspan region, *Aedes* were found at only one site, Buffalo pan (Table 1, p015kimgrsp), where adult mosquitoes where collected. This wetland was a small, shallow waterhole, only 5 m in diameter, formed from highly localised clay. Besides the vegetation, the other major difference between Holpan/Graspan (Table 1, p015kimgrsp) and the other sites is the geology and the derived soils. The rocks consist of amygdaloidal Andesite larva, geologically, very different from the Free State sediments of Karoo Supergroup. The Andesite forms red, iron-rich, soils with low clay-content, unlike the high, clay-content soils formed by the Karoo Supergroup sediments.

### Syntaxonomic description Community 1.

Community 1, *Eragrostis bicolor*, semi-arid grassland, is defined by the single grass species and consists of 27 relevés, contains 26 species varying from 1 – 9 per plot, and a total species-composition of 41 species, of which 34 form the synoptic table and represents the community of second-lowest species richness. The vegetation is dominated by the low, decumbent, drought-tolerant grass *Eragrostis bicolor* with a 92.6% consistency, and, *E. obtusa* (40.7% consistency), which are both species indicative of adaptation to arid areas. Both *Eragrostis* species are not found in any of the other seven wetland communities to the east. Additionally no *Juncus* or *Typha* were found with almost a complete absence of all sedges, with the exception of one relevé where *Schoenoplectus muricinux* occurred with a value of ‘+’. The alien, invasive weed, *Schkuhria pinnata*, has the second greatest cover/abundance in sub-community 1.4 and variant 1.5.1, it is also found in sub-community 1.1 and 1.5.2, but has not been used in the phytosociological nomenclature as its growth may vary rapidly from season to season. There is also a reluctance to use a weed (invasive alien species) in formal syntaxonomic nomenclature. Variant 1.5.2, has a number of wetland specialist species, including the two ephemerals, *Denekia capensis* and *Lobelia angolensis* with the low, spreading grass *Cynodon dactylon*, the wetland fern, *Marsilea capensis,* and the Obligate Wetland succulent, *Crassula natans*. All of which occurs in only one relevé with a value of ‘r’, but indicate habitat in transition to wetland, but still dominated by semi-arid grass species. The spreading forbs, *Chrysocoma ciliata* and *Geigeria filifolia* are species indicating arid conditions with shallow soils. No RFV livestock mortalities where recorded from this area, but adult *Aedes* were caught on one plot at this site.

Community 1 has 5 sub-communities and two variants:

Sub-community 1.1, *Tragus berteronianus*  *̶*  *Cynodon dactylon*, are both low, spreading grasses with 100% cover and consistency. Sub-community 1.2, *Geigeria filifolia ̶*   *Eragrostis bicolor*, semi-arid grassland, has an absence of most other species with *Eragrostis bicolor* having 83% abundance. Sub-community 1.3, *Falkia oblonga - Urochloa panicoides*, has the lowest cover of *Eragrostis bicolor* and the second highest species richness [14], including the only sedge in the entire Community 1, *Schoenoplectus muricinux*, low, spreading Amaranthaceae, *Alternanthera nodiflora*, and the single-stemmed, clover-leaf-like, wetland fern *Marsilea capensis* showing wetland affinities, represents the wettest conditions of Community 1.

Sub-community 1.4, *Eragrostis bicolor*, semi-arid grassland, has the lowest cover/abundance and total species richness of all sub-communities. The low species richness coupled with spiny shrub *Lycium cinereum*, and *Eragrostis plana* indicate not only the driest of the wetland sub-communities, but a change to upland conditions. Sub-community 1.5, *Stachys hyssopoides*  *̶*   *Eragrostis bicolor*, semi-arid grassland has two variants. The presence of the three shrubs *Stachys hyssopoides, Nidorella hottentotica, Selago alba* and the invasive weed, *Schkuhria pinnata* indicates a shallow-pan, with low clay-content soils, highly disturbed due to grazing and in transition from wetland vegetation to upland species. Variant 1.5.2 has two indicator species, the low, spreading *Lobelia angolensis* and the minute flowering *Denekia capensis* both ephemerals and habitat specialists of vernal conditions (Species group H, S1 Appendix).

### Ecological parameters Communities 2, 3 and 4.

Community 2, 3 and 4 are a mix of low, spreading grasses (*Cynodon dactylon*) and rounded, thin-stemmed sedges and include the upland, shallow depressions at which floodwater *Aedes* have been collected on an on-going basis (Alan Kemp, pers. com. 2014), and represent wetlands at the centre of high RVFV-related livestock mortality levels. Community 2, *Cyperus laevigatus ̶ Agrostis lachnantha*, sedge/grass wetland is defined by *Agrostis lachnantha* and would conform with Geldenhuys’ ‘scrub and sedge pans’. Adult *Aedes* and *Culex* were caught and under suitable conditions, and occurred in large numbers in these communities, as well as some distance from wetlands under the legumes (Fabaceae), growing adjacent to the farm house (Mr. Kobus Steenkamp, pers. com. 2014). Arum et al. [23] mosquitoes selecting some plant species over others due to the variation of microclimate conditions under each plant and mosquitoes feeding on the sugars in plant sap. Mosquitoes also selected against plants which produce secondary metabolites which act as repellents [23].

The rush *Juncus rigidus*, is a habitat specialist, growing on sandy, halophytic soils, in association with other salt-tolerant plants. It is absent from communities 1, 2, 3 and 5, but is found in communities 4 (67% consistency) and 6 and dominates communities 7 and 8 where it has high cover/abundance (B-B values of 2-5) and consistency values (57%). Its presence indicates a gradient from low-saline, sandy soils to high-saline, clay soils in areas of relatively high rainfall.

### Syntaxonomic description Communities 2, 3, and 4.

Community 2, *Cyperus laevigatus ̶ Agrostis lachnantha*, sedge/grass wetland (12 relevés), is dominated by the rhizomatous, sedge *Cyperus laevigatus* (75% consistency) and the medium-height (50-80 cm) obligate wetland grass *Agrostis lachnantha* (50% consistency). There is a wetland, phytosociological association of *Agrostis lachnantha* with *Pseudognaphalium luteo-album* (S1 Appendix), a soft, grey Asteraceae obligate wetland species which occurs elsewhere in Free State wetlands [34] and accompanied with the sedge, *Isolepis cernua* (S1 Appendix) indicate wetlands capable of holding water for several months allowing for the establishment of more obligate wetland species. The limited presence of the two halophytic succulent shrubs, *Salsola kali*, and *S. glabrescens* (S1 Appendix), grow on salt-rich, high clay-content soils, indicated community 2 is a wetland community on the margins of endorheic salt pans.

Community 3, *Fuirena coerulescens ̶ Echinochloa colona*, sedge/grass wetland (11 relevés). The dominant species is the low (30cm), bright-green, cylindrical-stemmed sedge *Fuirena coerulescens,* found almost exclusively in community 3, and with the defining grass *Echinochloa colona* are species indicative of increased habitat wetness. These category 2 wetlands (Table 1 and Fig 4) include sites such as Weltevreden (Table 1, p002bulwltv), Lamarloo (Table 1, p004bullmrl) and Deelpan (Table 1, p013bradlpn) which recorded some of the highest Rift Valley fever livestock mortality rates during the 2010-11 outbreak. The vegetation growing at category 2 pans does not form dense, rhizomatous stands, but discrete, tufts of graminoids and forbs. As with the Deelpan site, fire will burn off all the above ground material and leave a bare pan (Fig 4). As the growing season progresses, the vegetation will eventually form dense, continuous cover, with some tall species (*Sorghum* hybrid, S1 Appendix) reaching 2m in height. The pan/wetlands on which Community 3 vegetation grows, are dry until filled by sufficient seasonal rain. They are not inundated long enough to prevent the growth of geophytes such as *Moraea polystachya* and *Albuca prasina*, which occur almost exclusively in Community 3.

Community 4, *Hemarthria altissima ̶ Schoenoplectus muricinux*, grass/sedge wetland is the smallest of all communities (3 relevés). The dominant species is the tall, perennial, mat-forming, grass *Hemarthria altissima* (100% consistency), and with the tall (50-100 cm), diagnostic species, *Schoenoplectus muricinux* (75% consistency), which is a perennial, rhizomatous, sedge, combined with the high cover/abundance values distinguishes this community. The decision to not use *Phyla nodiflora*, a prostrate, spreading forb, as the diagnostic species despite it have a 100% consistency, is based on its widely varying seasonal cover/abundance, coupled with it being a species which allows successional plant-development in open, shallow pan-depressions. It is the most species-poor community after Sub-communities 1.1, 1.2 and 1.3, with only 14 species (S1 Appendix). The rush, *Juncus rigidus* (S1 Appendix, Species Group U), is salt-tolerant, growing on sandy soils, and combined with the limited presence of *Eragrostis plana, Hibiscus trionum* and *Panicum coloratum*, may indicate a community in transition from sandy, moderately saline soils with lower inundation, indicative of FAC or FAW conditions, to status of OBL species found in permanent wetlands [36, 49].

### Ecological parameters Community 5.

Community 5 is comprised of a wetland species associated with an artesian spring arising from calcrete strata approximately 3m thick. This semi-permanent, fresh water stream is the habitat for the submerged aquatic plant, *Lagarosiphon major,* a rhizomatous, perennial which forms dense stands of closely-packed stems rooted in the mud. The flowers are minute, <1mm and located in the axials of the whorled leaves. Where the stream spreads out from the initial, 1-5 m wide confined, channel, the OBL succulent forb *Crassula natans*, the sedge, *Eleocharis dregeana* and the submerged geophyte *Limosella grandiflora* grow. They are habitat specialists found only in community 5 and select for semi-permanent freshwater wetlands [34].

### Syntaxonomic description Community 5.

Community 5, *Cyperus laevigatus ̶ Pseudschoenus inanis*, sedge wetland (8 relevés, 28 species), and is the wettest of all the associations, demonstrated by the submerged aquatic *Lagarosiphon major* and the OBL, *Crassula natans*, *Eleocharis dregeana*, *Limosella grandiflora,* and *Rorippa nasturtium-aquaticum* (S1 Appendix). The dominant species *Cyperus laevigatus* (88% consistency) is a medium-tall (40-60 cm), densely-rhizomatous sedge. The defining species *Pseudschoenus inanis* (50% consistency), is tall (100-200 cm), densely-rhizomatous sedge, with cylindrical, ridged culms. Both sedge species select for brackish water, moderately alkaline, low clay-content soils, occurring in wet, saline or alkaline habitats. Community 5 wetlands are found as upland depressions (Dealsville: p009deaqwgg) in areas of reported high RFV livestock mortality. *Culex* mosquitoes where found but no *Aedes*.

### Ecological parameters Community 6.

Community 6 is composed of fresh-water wetland vegetation found mostly at the Seven Dams area, occurring on low clay-content soils and from which no RVF infections were reported. The vegetation is dominated by the sedge *Cyperus longus* and has a significant grass component as well as numerous OBL forbs and geophyte species. From this study, community 6, *Agrostis lachnantha ̶ Cyperus longus*, grass/sedge wetland, is dominated by the OBL grass *Agrostis lachnantha*, a thin, medium-tall (60-100cm) grass, and conforms to Geldenhuys’ [25], ‘mixed grass pans’.

### Syntaxonomic description Community 6.

Community 6, *Agrostis lachnantha ̶ Cyperus longus*, grass/sedge wetland (17 relevés), has the dominant sedge, *Cyperus longus* (72% consistency), and diagnostic OBL grass, *Agrostis lachnantha* (53% consistency). Both of these OBL species are freshwater habitat specialists selecting for low-velocity or still water. Community 6 is the most species rich of all the associations with a total of 58 species, of which 29 occur in 1 or 2 relevés. *Cyperus longus* is a medium height (40-70cm), spreading rhizomatous sedge which shares a freshwater habitat preferences with sandy soils of low clay-content, and high organic content with other OBL species. This includes the OBL forb *Rumex lanceolatus* (65% consistency), and has the greatest presence in all communities. These are palustrine wetlands, seasonally inundated, with low-clay soils, but also having anaerobic wetland conditions due to lengthy periods of inundation. Most of Community 6 wetlands occur on the dolerite found at 7 Dams Conservancy, Bloemfontein and are linear structures, no pans occur as with other sites. In 2010, no RVF mortalities were reported from any of Community 1 or 6 sites. This may be an indication of the difference in geology and thus soils.

### Ecological parameters Communities 7 and 8.

Communities 7 and 8 are dominated by sedges: *Cyperus laevigatus, Cyperus marginatus, Scirpoides dioecious,* and *Juncus rigidus*. These communities are near to the wetland sites where the highest Rift Valley fever mortality levels were reported. This includes the endorheic pan littoral zones with high salt-content soils. Additionally, for this study, Community 7, *Scirpoides dioecious ̶ Juncus rigidus*, sedge/*Juncus* wetland, has a lesser component of *Leptochloa fusca*, the currently accepted synonym for Diplachne, and identified as two of the 4 pan-types by Geldenhuys as ‘closed or open *Diplachne* pans’. The presence of the two morphologically different forms of the grass *Leptochloa fusca* – one creeping and the other erect, indicate upland depressions/palustrine wetlands with limited vegetation cover. The presence of the two halophytic species, *Sporobolus albicans* and the low, succulent wetland forb *Hypertelis salsoloides* indicates salinity, on moderate to high clay content soils, with standing water producing redox conditions found on endorheic saline pans characteristic of the western Free State. Swarms of mosquitoes were encountered on subsequent visits. The farmer owner at De Dam (p006bftddmm) did not report livestock deaths in 2010, but there were a moderate number of sheep deaths and one human fatality in the district. Included in these wetland types of Community 7 are the anthropogenic wetlands created by pivot irrigation which produce saline soils found at Rooibokkamp (p010jacrtrv) and Waterput (p007lucwtrp) near Jacobsdal (p010jacrtrv) and Luckhoff (p007lucwtrp) respectively in the far western Free State.

### Syntaxonomical description Community 7.

Community 7, *Scirpoides dioecious ̶ Juncus rigidus*, sedge/*Juncus* wetland, is dominated the tall, densely-tufted sedge, *Scirpoides dioecious* (88% consistency) and defined by rush, *Juncus rigidus* (59% consistency), a habitat specialist for sandy, hyaline soils. It comprises 17 relevés, and with the exclusive presence of the bunch-grass *Leptochloa fusca*, with the distinctive red-pattern on the leaves, coupled with the highest cover/abundance and consistency in all communities of both *Scirpoides dioecious* (83%), and *Juncus rigidus* (71%). This, together with the almost complete absence of *Cyperus laevigatus*, Community 7 indicates environmental and ecological parameters sufficiently different to justify naming in a separate community, distinct from Community 8. The wetland vegetation forms associations found at open depressions on sandy or moderate clay soils at Petrusburg (p011petmrtn), and Oppermansgronde (p008oppdmsh). Moderate to high mortality rates were recorded from these sites during the 2010-11 outbreak.

### Syntaxonomical Description Community 8.

Community 8, *Cyperus laevigatus ̶ Juncus rigidus*, sedge/*Juncus* wetland vegetation, is found along pan margins but generally not in the main areas of permanent to semi-permanent inundation. It has two sub-communities.

Community 8, *Cyperus laevigatus ̶ Juncus rigidus*, sedge/*Juncus* wetland, is the largest of all the communities and comprised of 32 relevés, 58 species (31 species in 2 or 1 relevés which do not contribute ecological significance to species groups), with two sub-communities. Livestock mortalities were recorded throughout Community 8 during the 2010 outbreak. The dominant species is *Cyperus laevigatus* (84% consistency), and the diagnostic species is the rush *Juncus rigidus* (56% consistency). The species composition indicates the highest saline conditions of all wetlands, with high clay-content soils. The presence of the weeds *Circum vulgare* (Scottish thistle), and the low, creeping Fabaceae *Trifolium repens* show high degrees of disturbance. *Scirpoides dioecious* (50% consistency) is a robust, spreading, tufted sedge with stiff tips, of medium height 60-80 cm, found on the margins of wetlands with saline soils, which does not grow in soils inundated for any length of time.

Sub-Community 8.1, *Cyperus marginatus ̶ Schoenoplectus triqueter*, sedge wetland, is a sedge community of 10 relevés, dominated by *Cyperus marginatus* (100% consistency) and the diagnostic species *Cyperus laevigatus* (70% consistency). The notable presence of the OBL sedge *Schoenoplectus triqueter* and *Rorippa nasturtium-aquaticum* (watercress, found in flowing, freshwater) indicates moderate to high clay-content soils with significant organic component found on the margins of endorheic salt pans such as Lamarloo (p004bullmrl) near Builtfontein. The sedge *Schoenoplectus triqueter* is uncommon, found at only two sites; Lamarloo (p004bullmrl) and Deelpan (p013bradlpn), both sites recording some of the highest reports of livestock mortalities during the 2010-11 outbreak. *Schoenoplectus triqueter* has stout, discrete rhizomes, and a distinctive, broad, 3-sided, concave, leaf with a pointed terminal inflorescent. Prior to this study there were only two vouchers at the UFS Herbarium, indicating it has limited occurrence in the Free State.

Sub-Community 8.2, *Scirpoides dioecious ̶ Hypertelis salsoloides*, sedge/succulent forb wetland, (22 relevés), is defined by the sedge *Scirpoides dioecious* (50% consistency), and despite using *Hypertelis salsoloides*, a low, spreading forb occurring with values of ‘+’ and ‘r’ (41% consistency), as a diagnostic species, it is a habitat-specialist indicative of open, shallow wetland depressions (water depth < 30 cm), which have grey clay soils and which are rain-fed. Petrusburg (p011petmrtn) is one such characteristic site. These sites include Oppermansgronde (p008oppdmsh) and the anthropogenic wetlands at Jacobsdal (p010jacrtrv) and Luckhoff (p007lucwtrp). Due to the constant irrigation, the soils are now saline soils with low clay content. Medium to low mortality rates were recorded during the outbreak. Limited numbers of *Culex* adults and larvae were collected but no *Aedes* were detected.

The yellow dots indicate the farm names on which the study sites are found.

**References**

1. Truswell JF. An Introduction to the Historical Geology of South Africa. Purnell Press, London; 1970.

2. McCarthy T, Rubidge B. The story of Earth and Life. Struik Publishing, Cape Town; 2005.

3. Bezuidenhout H, Bradshaw P. Vegetation landscapes of Mokala National Park (Northern Cape). Unpublished Internal Report for SANParks, Scientific Services, Kimberley; 2013.

4. Bezuidenhout H. The classification, mapping and description of the vegetation of the Rooipoort Nature Reserve, Northern Cape, South Africa. Koedoe 2009;51(1):1-11.

5. Verster M. Wetland soil properties conducive to the aestivation of Rift Valley fever infected Aedes mosquitos in central South Africa (MSc thesis). Department of Agriculture and Soil Science, University of the Free State: South Africa; 2016.

6. Idema SWJ, Jacobs EO , editors. Land Types of the Map, 2824 Kimberley, 2826 Winburg, 2924 Koffiefontein, 2629 Bloemfontein. Memoirs on the Agricultural Natural Resources of South Africa, Number 14, ARC-institute for soil, climate and water, Pretoria; 2012.
